# Machine Learning-Based Prediction of Unplanned Readmission Due to Major Adverse Cardiac Events Among Hospitalized Patients with Blood Cancers

**DOI:** 10.1177/10732748251332803

**Published:** 2025-04-17

**Authors:** Nguyen Le, Sola Han, Ahmed S. Kenawy, Yeijin Kim, Chanhyun Park

**Affiliations:** 1 Health Outcomes Division, College of Pharmacy, 15528 The University of Texas at Austin, Austin, TX, USA

**Keywords:** blood cancer, cardiovascular diseases (CVDs), cancer, hematology, readmission, machine learning (ML)

## Abstract

**Background:**

Hospitalized patients with blood cancer face an elevated risk for cardiovascular diseases caused by cardiotoxic cancer therapies, which can lead to cardiovascular-related unplanned readmissions.

**Objective:**

We aimed to develop a machine learning (ML) model to predict 90-day unplanned readmissions for major adverse cardiovascular events (MACE) in hospitalized patients with blood cancers.

**Design:**

A retrospective population-based cohort study.

**Methods:**

We analyzed patients aged ≥18 with blood cancers (leukemia, lymphoma, myeloma) using the Nationwide Readmissions Database. MACE included acute myocardial infarction, ischemic heart disease, stroke, heart failure, revascularization, malignant arrhythmias, and cardiovascular-related death. Six ML algorithms (L2-Logistic regression, Support Vector Machine, Complement Naïve Bayes, Random Forest, XGBoost, and CatBoost) were trained on 2017-2018 data and tested on 2019 data. The SuperLearner algorithm was used for stacking models. Cost-sensitive learning addressed data imbalance, and hyperparameters were tuned using 5-fold cross-validation with Optuna framework. Performance metrics included the Area Under the Receiver Operating Characteristics Curve (ROCAUC), Precision-Recall AUC (PRAUC), balanced Brier score, and F2 score. SHapley Additive exPlanations (SHAP) values assessed feature importance, and clustering analysis identified high-risk subpopulations.

**Results:**

Among 76 957 patients, 1031 (1.34%) experienced unplanned 90-day MACE-related readmissions. CatBoost achieved the highest ROCAUC (0.737, 95% CI: 0.712-0.763) and PRAUC (0.040, 95% CI: 0.033-0.050). The SuperLearner algorithm achieved slight improvements in most performance metrics. Four leading predictive features were consistently identified across algorithms, including older age, heart failure, coronary atherosclerosis, and cardiac dysrhythmias. Twenty-three clusters were determined with the highest-risk cluster (mean log odds of 1.41) identified by nonrheumatic/unspecified valve disorders, coronary atherosclerosis, and heart failure.

**Conclusions:**

Our ML model effectively predicts MACE-related readmissions in hospitalized patients with blood cancers, highlighting key predictors. Targeted discharge strategies may help reduce readmissions and alleviate the associated healthcare burden.

## Plain Language Summary

Patients with blood cancers, like leukemia, lymphoma, and myeloma, often undergo treatments that can harm their hearts, leading to an increased risk of serious heart problems. These issues can result in unplanned hospital readmissions, which place significant strain on both patients and healthcare systems. Predicting and preventing these readmissions could improve patient care and reduce healthcare costs.

We used data from a large, nationwide database to study patients with blood cancers who were hospitalized between 2017 and 2019. We focused on predicting whether these patients would be readmitted to the hospital within 90 days due to serious heart problems (heart attacks, strokes, or heart failure). We tested six different machine learning (ML) algorithms to see which one was best at making these predictions. The algorithms were trained on data from 2017-2018 and tested on 2019 data. We also used a method called “SuperLearner” to combine the best features of all six algorithms to improve accuracy.

Among 76 957 patients, 1.3% had an unplanned readmission within 90 days due to serious heart problems. The most accurate algorithm was CatBoost, which correctly identified a significant portion of these cases. The SuperLearner method improved predictions. Key factors that increased the risk of readmission included older age, heart failure, diabetes, and certain heart conditions. We also identified a high-risk group of patients with specific heart valve disorders and heart failure.

This study shows that ML models can help predict which patients with blood cancers are at the highest risk of unplanned readmission due to serious heart problems. With this knowledge, healthcare providers can develop better discharge plans, potentially reducing readmissions and lowering healthcare costs.

## Introduction

In the United States (US), blood cancers, including leukemia, lymphoma, multiple myeloma, myeloproliferative neoplasms, and myelodysplastic syndromes, account for more than 9% of annual cancer cases, with approximately 20 new cases diagnosed every hour.^1^ Specifically, leukemia accounts for 3%, lymphoma for 4.5%, and multiple myeloma for 1.8%.^1^ Survival rates for blood cancers have improved significantly over the past decade, likely due to early detection (e.g., advanced blood tests and genetic profiling) and the availability of novel treatments (e.g., tyrosine kinase inhibitors (TKIs) and chimeric antigen receptor (CAR) T-cell therapy).^2,3^

Along with these improvements, a substantial financial burden of blood cancer care on both the healthcare system and patients has been documented.^4-6^ Health care costs for blood cancer are potentially driven by hospital inpatient utilizations. Studies commissioned by the Leukemia & Lymphoma Society Hospital (LLS) showed that hospital admissions accounted for the largest portion of 24-month spending after diagnosis.^4,5^ Patients with blood cancers had the highest average cost per stay and the longest average length of stay among the top 20 most common types of cancer, according to a report from the Healthcare Cost and Utilization Project (HCUP).^7^ Moreover, unplanned readmissions, which significantly contribute to health care costs, are high among patients with blood cancers.^8^ A systematic review of hospital admissions among patients with cancer in the US revealed that patients with blood cancer – along with bladder, pancreatic, and ovarian cancers – had the highest readmission rates compared to other cancer types.^9^ High rates of unplanned readmissions also impose an additional burden on patients and their families.^10^ Therefore, preventing avoidable readmissions could improve patient quality of life as well as alleviate financial burdens.^8,11^

Cardiovascular (CV) related unplanned readmissions in patients with cancer have emerged as a significant challenge for healthcare providers. A previous study estimated that 35% of patients with cancer experience an unplanned hospitalization within the first year after cancer diagnosis, of which 5.8% are due to CV reasons.^12^ Patients with cancer also had higher CV-related hospitalization and unplanned readmission rates due to cardiovascular disease (CVD) compared to those without cancer.^13,14^ Recent evidence showed that blood cancers were associated with an increased risk of incident CVDs, such as ischemic heart disease, stroke, atrial fibrillation, heart failure, cardiomyopathies, and CV-related mortality,^15^ which are known as a high-risk factors for hospital readmission.^16,17^ Therefore, concerns about CV-related readmissions among this population need more attention.

Previous studies have focused on assessing readmission rates after stem cell transplantation,^18,19^ and determining predictors for unplanned readmissions in patients with blood cancers.^20-22^ Given the complex conditions of patients with blood cancers, traditional methods (e.g., logistic regression) are typically limited to linear relationships and may not capture interactions between predictors. Conversely, machine learning (ML) based methods, which relax assumptions about linear relationships and allow for the modeling of complex interactions, have been potential tools to help reduce hospital readmissions.^23,24^ Several ML models have been developed for predicting hospital readmissions in the general population, showing better predictive performance compared to traditional methods (e.g., logistic regression) and conventional methods (e.g., LACE score).^24-26^ While conventional methods offer simplicity in calculation and interpretation, they often sacrifice predictive performance. There has been an increasing number of studies on the development of ML-based readmission prediction models in patients with cancer.^27-31^ However, only a few studies have focused on developing ML models for predicting readmissions in patients with blood cancers.^32^

To our knowledge, no study has developed ML models for predicting CV-related unplanned readmissions in patients with blood cancers. Existing studies have typically used narrow timeframes (e.g., 30 days), which may not fully capture CV-related readmissions or account for the elevated CVD risk in cancer patients.^33-36^ In this study, we extended the prediction period to 90 days to better capture CV-related readmissions. Additionally, ML-based methods were used to address the complex interactions among characteristics of patients with blood cancers. The objective of this study was to develop an ML model to predict 90-day unplanned readmissions due to major adverse cardiovascular events (MACE) among hospitalized patients with blood cancers. We further identified predictive factors and high-risk subgroups for 90-day unplanned MACE readmission.

## Methods

### Data Source

We used the Nationwide Readmissions Database (NRD) from 2017 and 2018 for training (training set) the machine learning algorithms, and the 2019 NRD for testing (testing set) the performance of developed algorithms. The NRD, a part of the Healthcare Cost and Utilization Project (HCUP), provides nationally representative information for approximately 60% of all US hospitalizations across 28 states. The NRD contains demographics, primary payers, household income, admission diagnoses and procedures, and lengths of stay.^37^ The NRD has been used to develop machine learning-based prediction models for readmissions in patients with various cancers, including spinal, esophageal, or any cancer types.^38-40^ Since the NRD is publicly available deidentified data, the Institutional Review Board of The University of Texas at Austin exempted the study and informed consent was not required.

### Study Design

We conducted a population-based cohort study following the Transparent Reporting of a Multivariable Prediction Model for Individual Prognosis or Diagnosis (TRIPOD) guidelines and the Guidelines for Developing and Reporting Machine Learning Models in Biomedical Research.^41,42^ Patients could not be tracked longitudinally because the NRD provides yearly data, and each year, patients may have been admitted multiple times. We selected the earliest hospitalization as the index hospitalization for each patient in each year. We determined patient baseline characteristics (features) based on the information retrieved from the index hospitalization. We tracked subsequent hospitalizations following the index hospitalization of each patient to identify the readmission outcome. If a patient had multiple readmissions that met the definition, we selected only the closest readmission to the index hospitalization.

### Study Population

**Figure 1.**
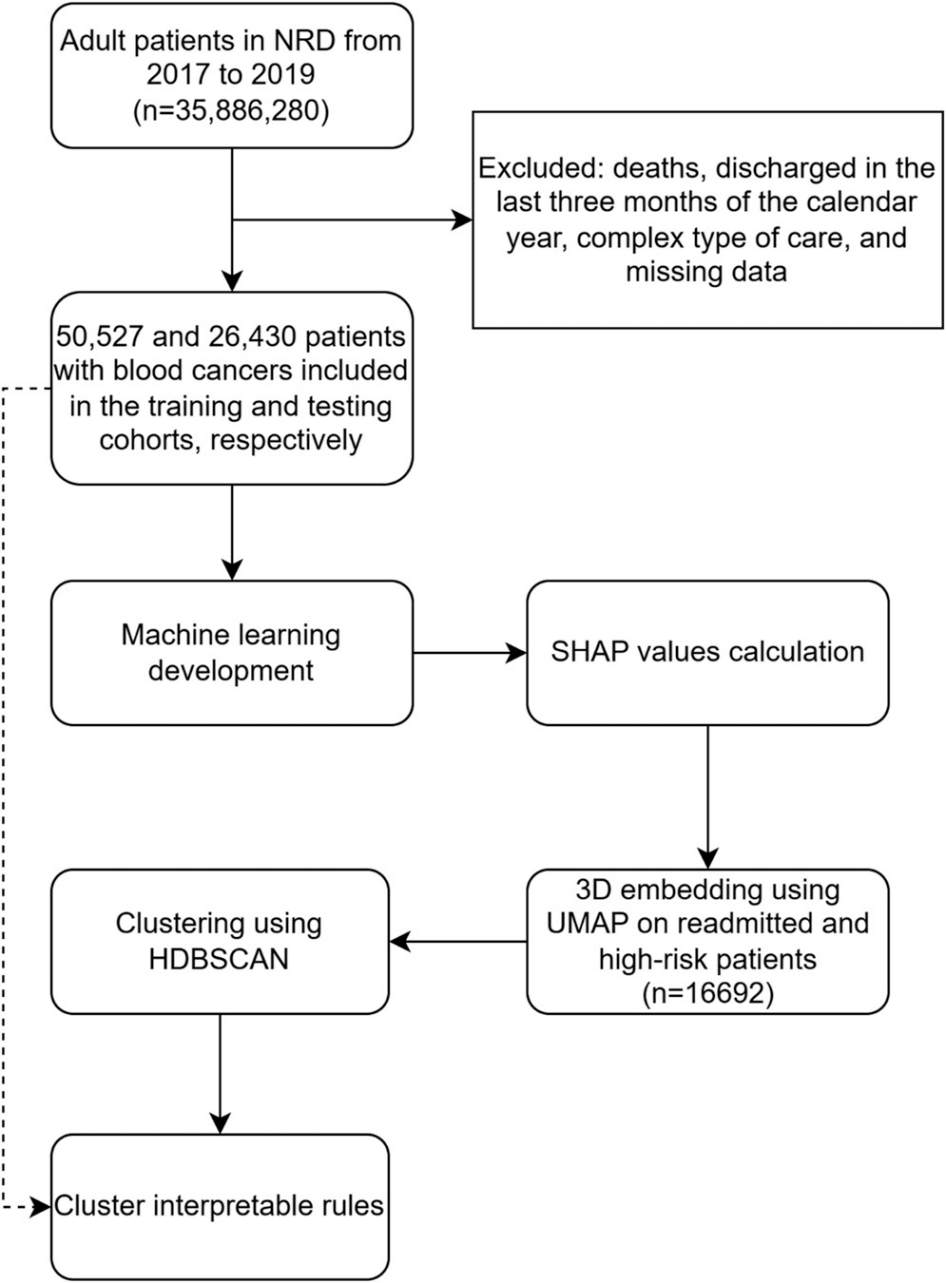
Study Flowchart. Caption: Patients Aged 18+ With a Primary Diagnosis of Blood Cancers (Leukemia, Lymphoma, Multiple Myeloma, Myelodysplastic Syndrome) Were Included. Exclusions Were Patients Who Died, Experienced Complex Care (e.g., Transfers or Combined Same-Day Stays), Were Discharged in Fourth Quarter, or had Missing Data. Data From 2017-2018 Were Used to Train Seven Machine Learning Models, With 2019 Data for Testing. SHAP Analysis was Performed for Model Interpretation, and High-Risk Patients (Predicted Readmission Risk >75th Percentile) Were Identified. SHAP Values for These Patients Were Used for Clustering Analysis, and Decision Rules Were Applied to Link Original Dataset Information With SHAP-Based Clusters for Interpretable Results. Note: NRD: National Readmission Database; SHAP: SHapley Additive exPlanations; HDBSCAN: Hierarchical Density-Based Spatial Clustering of Applications With Noise.

We included patients aged 18 years and older diagnosed (primary diagnosis) with blood cancers, including leukemia, lymphoma, multiple myeloma, and myelodysplastic syndrome, using HCUP Clinical Classifications Software Refined (HCUP-CCSR) diagnosis codes (Supplemental Table 1). We excluded patients who died, experienced complex types of care (e.g., transfers and same day stays combined) in the index hospitalization, those discharged in October, November, or December, and those with missing information (Figure 1).

### Measures

#### Outcome

Our primary outcome was a 90-day unplanned MACE hospital readmission, defined as a nonelective readmission that occurred within 90 days of the index hospitalization due to MACE. MACE was a composite of (1) acute myocardial infarction, (2) acute coronary syndrome, (3) heart failure, (4) stroke and transient ischemic attack, (5) revascularization procedures, (6) malignant arrhythmias and (7) cardiovascular mortality.^43,44^ We determined the MACE using the HCUP-CCSR and International Classification of Diseases-10^th^ Revision Procedure Coding System (ICD-10-CM) codes for diagnosis and procedures (Supplemental Table 1).

#### Features (Predictors)

We included demographics, admission and discharge details, and clinical features as candidate predictors. The demographics included age, sex, resident status, primary insurance payer, urban-rural location, and median household income. The admission and discharge details included length of stay, discharge month and quarter, weekend admission, and discharge disposition. The clinical features were retrieved from 542 HCUP-CCSR diagnoses, and 326 HCUP-CCSR procedures.^45,46^ The CCSR diagnoses and procedures are generated to aggregate ICD-10-CM codes into clinically meaningful categories and computationally efficient for learning.^47^ We selected only those predictors that had a number of events per variable larger than 5 in both the readmitted and non-readmission groups.^48^ Categorical features, including health insurance, urban-rural location, discharge month and quarter, discharge disposition, and household income, were encoded using one-hot encoding (with reference level), while continuous features were used in their original form. All included predictors are shown in Supplemental Table 2. Missing data proportions in predictors, which are urban-rural location and discharge disposition, were less than 1%; therefore, we conducted a complete case analysis by excluding these cases.

### Machine Learning Model Development

We used six ML algorithms for developing the prediction model: (1) Logistic regression with L2 regularization (LR-L2), (2) Support Vector Machine (SVM), (3) Complement Naïve Bayes (CNB), (4) Random Forest (RF), (5) Extreme Gradient Boosting (XGBoost), and (6) Categorical Boosting (CatBoost). The first five algorithms were commonly used for predicting hospital readmissions.^40,49,50^ While CatBoost is well-suited for dealing with categorical data, it accounted for a vast majority of features in our study.^51^ Additionally, we constructed a two-layer stacking model using the Super Learner algorithm.^52,53^ In this algorithm, the first layer (base-layer) contained models, which performed not statistically different on classification metrics (defined below) and the second layer (meta-layer) was logistic regression without regularization with 5-fold stacking process. The data in Table 1 highlighted a significant imbalance in outcomes, with the readmitted group comprising only 1.34% of cases. To address this, we applied the cost-sensitive learning approach using class weights.^54-56^ To optimize algorithm performance, we tuned the value of class weights ranging from 0.5 to 1.5 times the imbalanced ratio.^57^

**Table 1.** Baseline Characteristics of Study Population.

Characteristics	MACE-readmission (*n* = 1031)	No MACE-readmission (*n* = 75926)	SD	Training cohort (*n* = 50527)	Testing cohort (*n* = 26430)	SD
Age (years), mean (SD)	71.34 (12.80)	63.06 (16.17)	0.57	63.01 (16.19)	63.49 (16.09)	−0.03
Sex (Female), n (%)	423 (41.03)	32 929 (43.37)	−0.05	21 944 (43.43)	11 408 (43.16)	0.01
Resident, n (%)	979 (94.96)	68 335 (90.00)	0.19	45 522 (90.09)	23 792 (90.02)	0.00
Length of stay (days), Median (IQR)	7.0 (4.0-15.0)	9.0 (4.0-18.0)	−0.09	9.0 (4.0-18.0)	9.0 (4.0-4.0)	−0.01
Weekend admission, n (%)	193 (18.72)	12 691 (16.71)	0.05	8371 (16.57)	4513 (17.08)	−0.01
Health insurance, n (%)						
Medicare	742 (71.97)	38 188 (50.30)	0.46	25 437 (50.34)	13 493 (51.05)	−0.01
Medicaid	81 (7.86)	8514 (11.21)	−0.11	5682 (11.25)	2913 (11.02)	0.01
Private	181 (17.56)	25 317 (33.34)	−0.37	16 856 (33.36)	8642 (32.70)	0.01
Others (Self-pay, No charge, other)	27 (2.62)	3907 (5.15)	−0.13	2552 (5.05)	1382 (5.23)	−0.01
Urban-rural location, n (%)						
“Central” counties >=1 million population	326 (31.62)	23 327 (30.72)	0.02	15 704 (31.08)	7949 (30.08)	0.02
“Fringe” counties >=1 million population	284 (27.55)	20 694 (27.25)	0.01	13 767 (27.25)	7211 (27.28)	−0.00
Counties of 250 000-999,999 population	201 (19.50)	15 775 (20.78)	−0.03	10 360 (20.50)	5616 (21.25)	−0.02
Counties of 50 000-249,999 population	81 (7.86)	6671 (8.79)	0.01	4429 (8.77)	2335 (8.83)	−0.00
Micropolitan counties	81 (7.86)	5345 (7.04)	0.03	3550 (7.03)	1876 (7.10)	−0.00
Not metropolitan or micropolitan counties	46 (4.46)	4114 (5.42)	−0.05	2717 (5.38)	1443 (5.46)	−0.00
Household income, n (%)						
Quartile 1	246 (23.86)	17 481 (23.02)	0.02	11 616 (22.99)	6111 (23.12)	−0.00
Quartile 2	247 (23.96)	19 302 (25.42)	−0.03	13 118 (25.96)	6431 (24.33)	0.04
Quartile 3	273 (26.48)	19 724 (25.98)	0.01	12 942 (25.61)	7055 (26.69)	−0.02
Quartile 4	265 (25.70)	19 419 (25.58)	0.00	12 851 (25.43)	6833 (25.85)	−0.01
Discharge disposition, n (%)						
Routine	606 (58.78)	50 304 (66.25)	−0.15	33 547 (66.39)	17 363 (65.69)	0.01
Transfer to short-term hospital	12 (1.16)	1553 (2.05)	−0.07	1026 (2.03)	539 (2.04)	−0.00
Skilled nursing/Intermediate care Facility	148 (14.35)	8289 (10.92)	0.10	5509 (10.90)	2928 (11.08)	−0.01
Home health care	262 (25.41)	15 338 (20.20)	0.12	10 157 (20.10)	5443 (20.59)	−0.01
Against Medical advice	<11	442 (0.58)	−0.04	288 (0.57)	157 (0.59)	−0.00
Comorbidities*, n (%)						
AIDS	<11	507 (0.67)	0.00	330 (0.65)	184 (0.70)	−0.01
Alcohol abuse	17 (1.65)	1410 (1.86)	−0.02	922 (1.82)	505 (1.91)	−0.01
Anemias due to other nutritional deficiencies	392 (38.02)	23 467 (30.91)	0.15	15 588 (30.85)	8271 (31.29)	−0.01
Autoimmune conditions	42 (4.07)	2320 (3.06)	0.06	1551 (3.07)	811 (3.07)	0.00
Chronic blood loss (iron deficiency)	17 (1.65)	737 (0.97)	0.06	496 (0.98)	258 (0.98)	0.00
Leukemia	379 (36.76)	24 886 (32.78)	0.08	16 385 (32.43)	8880 (33.60)	−0.02
Lymphoma	555 (53.83)	47 932 (63.13)	−0.19	32 006 (63.34)	16 481 (62.36)	0.02
Metastatic cancer	41 (3.98)	2719 (3.58)	0.02	1716 (3.40)	1044 (3.95)	−0.03
Solid tumor without metastasis, in situ	<11	54 (0.07)	0.01	35 (0.07)	20 (0.08)	−0.00
Solid tumor without metastasis, malignant	166 (16.12)	6927 (9.12)	0.21	4640 (9.18)	2453 (9.28)	−0.00
Cerebrovascular disease	48 (4.56)	1929 (2.54)	0.11	1249 (2.47)	727 (2.75)	−0.02
Coagulopathy	495 (48.01)	38247 (50.37)	−0.05	25294 (50.06)	13448 (50.88)	−0.02
Dementia	33 (3.20)	2256 (2.97)	0.01	1477 (2.92)	812 (3.07)	−0.01
Depression	107 (10.39)	8035 (10.58)	−0.01	5308 (10.51)	2834 (10.72)	−0.01
Diabetes with chronic complications	191 (18.54)	8757 (11.53)	0.20	5662 (11.21)	3286 (12.43)	−0.04
Diabetes without chronic complications	145 (14.08)	7261 (9.56)	0.14	4968 (9.83)	2438 (9.22)	0.02
Drug abuse	13 (1.26)	1117 (1.47)	−0.02	730 (1.44)	400 (1.51)	−0.01
Heart failure	300 (29.10)	7184 (9.46)	0.51	4750 (9.40)	2734 (10.34)	−0.03
Hypertension, complicated	362 (35.11)	13 063 (17.20)	0.42	8543 (16.91)	4882 (18.47)	−0.04
Hypertension, uncomplicated	365 (35.40)	28320 (37.30)	−0.04	18 879 (37.36)	9806 (37.10)	0.01
Liver disease, mild	76 (7.37)	4822 (6.35)	0.04	3097 (6.13)	1801 (6.81)	−0.03
Liver disease and failure, moderate to severe	14 (1.36)	751 (0.99)	0.03	482 (0.95)	283 (1.07)	−0.01
Chronic pulmonary disease	194 (18.82)	10 335 (13.61)	0.14	6828 (13.51)	3701 (14.00)	−0.01
Neurological disorders affecting movement	<11	1308 (1.72)	−0.10	822 (1.63)	493 (1.87)	−0.02
Other neurological disorders	94 (9.12)	5593 (7.37)	0.06	3677 (7.28)	2010 (7.60)	−0.01
Seizures and epilepsy	22 (2.13)	1546 (2.04)	0.01	1024 (2.03)	544 (2.06)	−0.00
Obesity	151 (14.65)	9506 (12.52)	0.06	6258 (12.39)	3398 (12.86)	−0.01
Paralysis	20 (1.94)	1813 (2.39)	−0.03	1178 (2.33)	655 (2.48)	−0.01
Peripheral vascular disease	82 (7.95)	3225 (4.25)	0.16	2094 (4.14)	1213 (4.59)	−0.02
Psychoses	17 (1.65)	1537 (2.02)	−0.03	1000 (1.98)	554 (2.10)	−0.01
Pulmonary circulation disease	70 (6.79)	1975 (2.60)	0.20	1324 (2.62)	721 (2.73)	−0.01
Renal (kidney) failure and disease, moderate	169 (16.39)	8503 (11.20)	0.15	5575 (11.03)	3097 (11.72)	−0.02
Renal (kidney) failure and disease, severe	63 (6.11)	2729 (3.59)	0.12	1851 (3.66)	941 (3.56)	0.01
Hypothyroidism	160 (15.52)	9561 (12.59)	0.08	6280 (12.43)	3441 (13.02)	−0.02
Other thyroid disorders	17 (1.65)	1347 (1.77)	−0.01	873 (1.73)	491 (1.86)	−0.01
Peptic ulcer with bleeding	24 (2.33)	1274 (1.68)	0.05	847 (1.68)	451 (1.71)	−0.00
Valvular disease	147 (14.27)	3657 (4.82)	0.33	2455 (4.86)	1349 (5.10)	−0.01
Weight loss	185 (17.94)	13718 (18.07)	−0.01	8871 (17.56)	5032 (19.04)	−0.04
90-day MACE unplanned readmission				641 (1.27)	390 (1.47)	−0.02

Note: SD: Standardized difference; *Defined by HCUP Elixhauser comorbidities.

We tuned hyperparameters with stratified 5-fold cross-validation using the Optuna framework with 200 trials on the training set to optimize the $F_{2}$ score.^58^ The $F_{2}$ score, a modified F score, is a harmonization score between precision and recall metrics, in which the recall metric is given more weight.^59^ With the optimization of this score, we aimed to develop an algorithm with the ability to capture as many true positives while minimizing false negatives as possible.^60^ The optimal hyperparameter values for each algorithm are reported in Supplemental Table 3.

### Statistical Analysis for Classification Performance

We evaluated the models on the testing set using 10-fold cross-validation. The performance metrics used were the Area Under the Receiver Operating Characteristics curve (AUROC), the Area Under the Precision-Recall curve (PRAUC), precision and recall scores, balanced accuracy, balanced Brier score and $F_{2}$ score. The balanced Brier score, which is the sum of stratified Brier scores for both positive and negative instances, is used to measure the quality of the class probabilities generated by a model, particularly suitable for imbalanced data.^61^ We used the DeLong test, permutation test, Friedman’s test, and corrected t-test to determine whether the differences in AUROC, PRAUC, $F_{2}$ score, and balanced Brier score were statistically significant, respectively.^62-64^ We adjusted the *P*-value threshold for multiple pairwise comparisons using the Finner’s correction and the Bonferroni-Dunn methods.^65^ We reported the mean and 95% confidence interval (95% CI) of the metrics from 10 iterations of 10-fold cross-validation.

### Explainability

We represented features’ importance score by calculating the SHapley Additive exPlanations (SHAP) values.^66^ We defined the adversity cutoffs for continuous features and visualized potential interactions among SHAP values using partial dependence plots. We conducted a clustering analysis on SHAP values to further examine subpopulations clustered by the predicted risk of readmission.^67^ The SHAP values of the highest AUC model were included in this analysis. Initially, Uniform Manifold Approximation and Projection (UMAP) was used to reduce the SHAP values of the entire data set into two dimensions for visualization purposes.^68^ Next, we applied UMAP to project the SHAP values into three dimensions for readmitted and high-risk patients, identified as those with a predicted risk of readmission above the 75^th^ percentile of the total population risk scores. The Hierarchical Density-Based Spatial Clustering of Applications with Noise (HDBSCAN) was used to cluster the three-dimensional UMAP embeddings.^69^ Finally, we used decision rules via the SkopeRules package to generate interpretable rules for the identified clusters in the previous step. UMAP embedding was computed using a local neighborhood (n_neighbors) of 200 data points, and a minimum distance (min distance) of zero. Additionally, we tuned the HDBSCAN hyperparameters to optimize the average silhouette coefficient.^70^ We set a maximum depth search of ten for the decision rules learning.

### Software and Packages

We performed all the analyses using Python version 3.11 and developed machine learning models with the sklearn package.^71,72^ We constructed the Super Learner model using mlens package.^73^ The statistical tests were performed using R using the scmamp package.^74^

## Results

### Population Characteristics

A total of 76 957 hospitalized patients with blood cancers between 2017 and 2019 were included in the study. Among them, 1031 (1.34%) patients who experienced unplanned readmission within 90 days due to MACE were identified. The readmitted group was older (71.34 vs 63.06 years, SD = 0.57) and had a higher frequency of Medicare coverage (71.97% vs 50.30%, SD = 0.46) than the no readmission group. Additionally, the readmitted group had a higher prevalence of several comorbidities, including heart failure (29.10% vs 9.46%, SD = 0.51), complicated hypertension (35.40% vs 17.20%, SD = 0.42), valvular disease (14.27% vs 4.82%, SD = 0.33), and malignant solid tumor (16.12% vs 9.12%, SD = 0.21) (Table 1). Other baseline characteristic differences between the two groups were generally small (SD < 0.2). Regarding the training and testing cohorts, the baseline characteristic differences were small (SD < 0.2). The 90-day readmission rate was 1.27% and 1.47% in the training and testing cohorts, respectively.

### Performance of Machine Learning Algorithms

**Figure 2.**
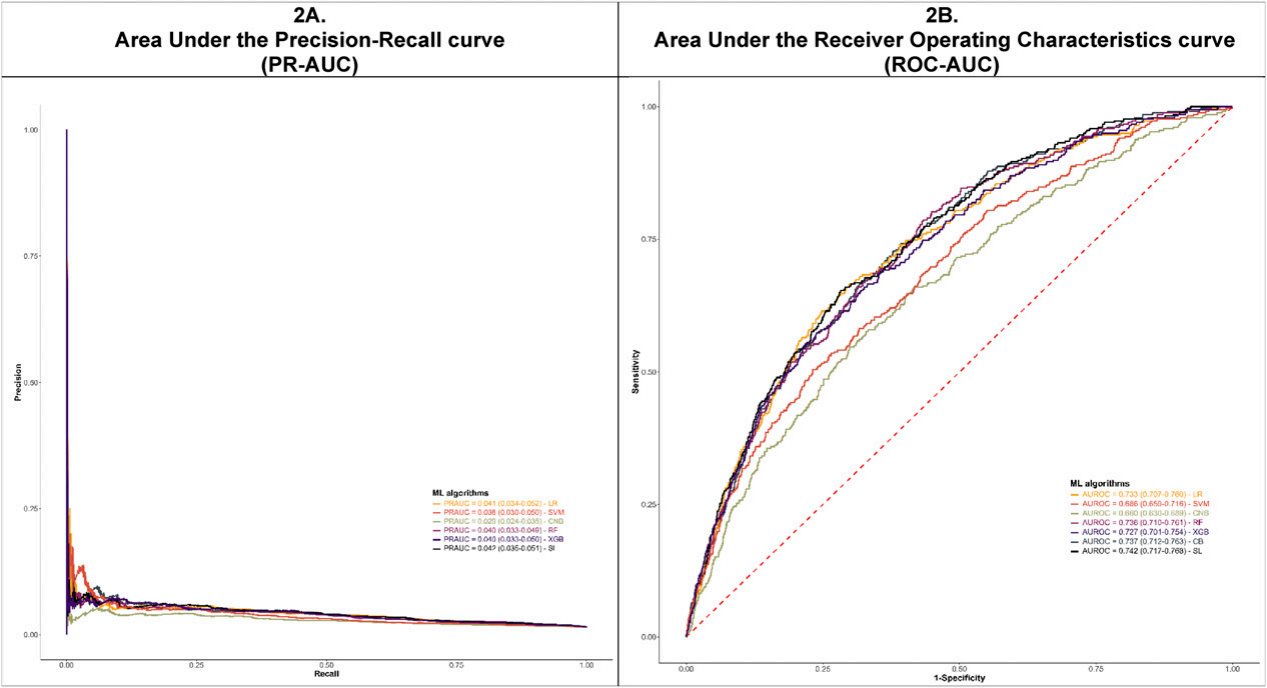
Predictive Performance of Developed Models. Caption: These figures present the Predictive Performance Using PR-AUC (Left) and ROC-AUC (Right) of Developed Models. The CatBoost Algorithm Achieved the Highest AUC Values for Both ROC (0.737, 95% CI: 0.712-0.763) and PR (0.040, 95% CI: 0.033-0.050). However, the Differences in ROC-AUC and PR-AUC Among Most ML Algorithms Were Not Statistically Significant, Except for the CNB Algorithm. The Stacking Algorithm (Super Learner) Achieved a Slight Enhancement in Performance Compared to the Single Algorithms in These Two Metrics. Note: CB: CatBoost; LR: Logistic Regression With L2 Regularization; CNB: Complement Naïve Bayes; RF: Random Forest; SL: Super Learner; SVM: Support Vector Machine; XGB: XGBoost.

Figure 2 and Table 2 show the classification performances of each ML algorithm on the testing cohort after training. The statistical tests for the metrics are presented in Supplemental Table 4. The predictive performance of the ML algorithms varied across metrics. The CatBoost algorithm achieved the highest AUC in both ROC and PR with values of 0.737 (95% CI, 0.712-0.763) and 0.040 (95% CI, 0.033-0.050), respectively. However, the differences in both ROC-AUC and PR-AUC across ML algorithms were mostly not statistically significant except for the CNB algorithm. On the other hand, the LR-L2 algorithm yielded the highest score of 0.1580 (95% CI, 0.1574-0.1582) in terms of $F_{2}$ score. The differences in $F_{2}$ score between the LR-L2 algorithm and the others were not statistically significant except for the CNB algorithm. Moreover, the RF algorithm performed best when considering the balanced Brier score as the evaluation metric, with a value of 0.4488 (95% CI, 0.4487-0.4488). Notably, the CNB and SVM algorithms performed significantly worse than the other algorithms in terms of AUCs (ROC and PR) and the balanced Brier score, respectively.

**Table 2.** Performance of Candidate Machine Learning Algorithms in Testing Cohort.

ML algorithms	*F*_*2*_ score	Precision	Recall	Balanced accuracy	Balanced Brier score
Logistic regression	0.1580 (0.1574-0.1582)	0.0456 (0.0454-0.0457)	0.4113 (0.4112-0.4114)	0.6414 (0.6413-0.6415)	0.4520 (0.4520-0.4521)
Support vector Machine	0.1407 (0.1404-0.1409)	0.0437 (0.0436-0.0438)	0.3162 (0.3161-0.3164)	0.6064 (0.6063-0.6064)	0.9529 (0.9529-0.9529)
Complement naïve bayes	0.1127 (0.1126-0.1128)	0.0269 (0.0269-0.0269)	0.5552 (0.5551-0.5553)	0.6275 (0.6275-0.6276)	0.5886 (0.5886-0.5888)
Random forest	0.1571 (0.1568-0.1573)	0.0455 (0.0454-0.0456)	0.4061 (0.4060-0.4063	0.6394 (0.6393-0.6395)	0.4488 (0.4487-0.4488)
XGBoost	0.1533 (0.1528-0.1535)	0.0451 (0.0449-0.0452)	0.3830 (0.3829-0.3831)	0.6310 (0.6309-0.6310)	0.4555 (0.4555-0.4556)
CatBoost	0.1528 (0.1523-0.1530)	0.0472 (0.0470-0.0473)	0.3470 (0.3469-0.3471)	0.6212 (0.6212-0.6212)	0.4554 (0.4553-0.4554)
Super learner	0.1651 (0.1467-0.1796)	0.0471 (0.0422-0.0514)	0.4422 (0.3910-0.4806)	0.6528 (0.6283-0.6719)	0.4509 (0.4377-0.4664)

Note: $F_{2}$ score (a modified F score), is a harmonization score between precision (positive predictive value) and recall (true positive rate) metrics, in which the recall metric is given more weight (twice weightage). The balanced Brier score, which is the sum of stratified Brier scores for both positive and negative instances, is used to measure the quality of the class probabilities generated by a model.

By leveraging the performance of four algorithms (i.e., LR-L2, RF, XGBoost, and CatBoost), the stacking algorithm (SuperLearner) achieved a slight enhancement in performance compared to the individual algorithms in most evaluation metrics, except for balanced Brier score. The values for ROC-AUC, PR-AUC, $F_{2}$ score and the balanced Brier score were 0.742 (95% CI, 0.717-0.768), 0.042 (95% CI, 0.035-0.051), 0.1651 (95% CI, 0.1467-0.1769), and 0.4509 (95% CI, 0.4377-0.4664), respectively.

### Feature Importance via SHAP Value Analysis

**Figure 3.**
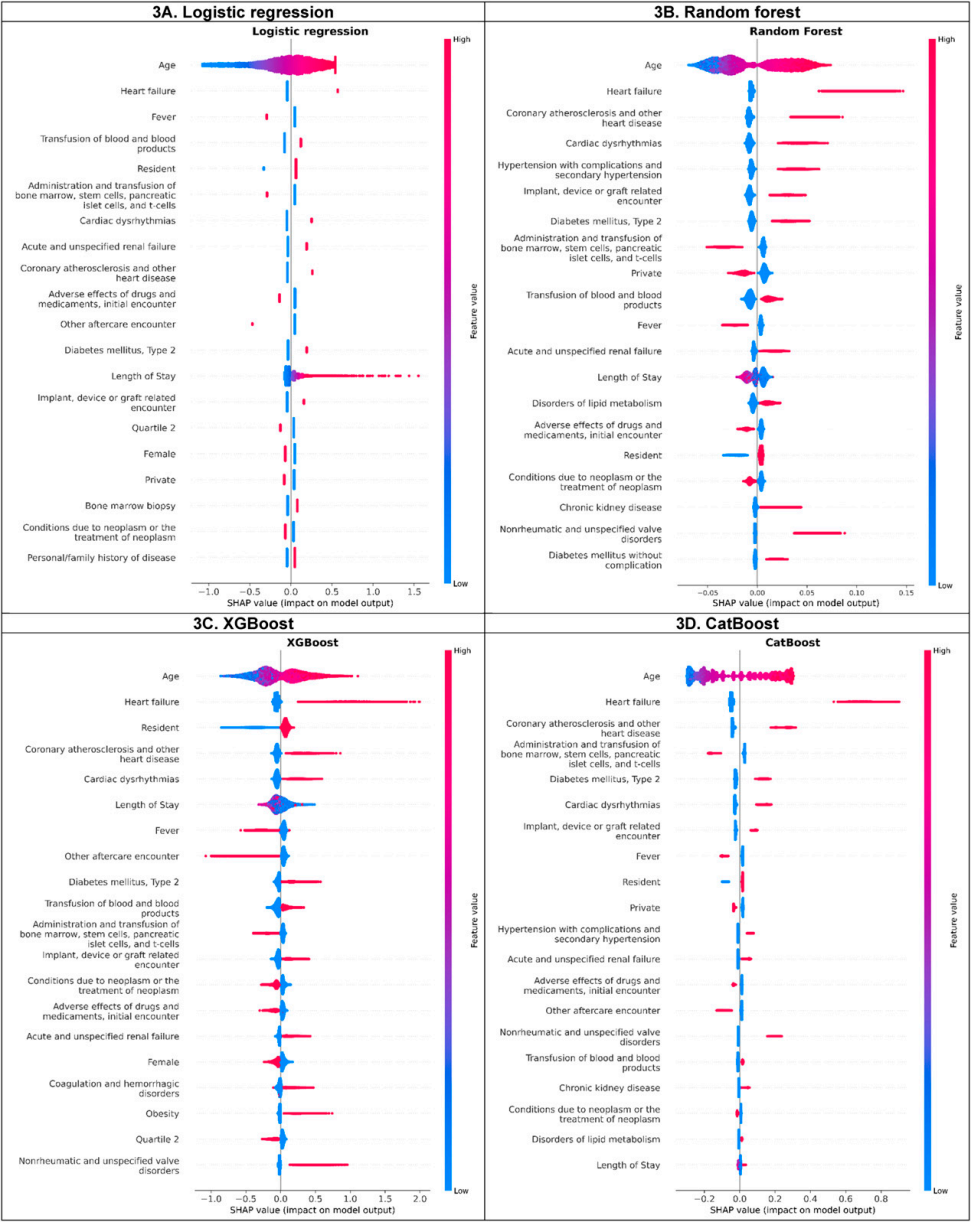
SHapley Additive exPlanations (SHAP) Value Plots of Models Developed by Algorithm. Caption: These figures present the SHAP Summary Plot for Logistic Regression, Random Forest, XGBoost, and CatBoost. The figures illustrate the Impact of Each Feature (Predictor) on and Highlight Key Features of the Predicted Risk of 90-Day Unplanned MACE Hospital Readmission. The x-Axis Represents the SHAP Values, which Indicate the Direction and Magnitude of Feature Contribution to the Outcome Prediction. The Feature Value (Gradient Color) on the Left y-Axis Reflects the Impacts of Increasing (Red/Pink Color) or Decreasing of the Predicted Risk Among Features.

Figure 3 displays the top 20 features with the highest SHAP values among the four best algorithms. Out of the 16 distinct features (extracted from the top 10 features of each model), four features were consistently identified across all four models. These were older age, presence of heart failure, coronary atherosclerosis and other heart diseases, cardiac dysrhythmias. Type 2 diabetes was among the top predictors for RF, XGBoost, and CatBoost but not for LR-L2.

SHAP analysis identified an interaction effect between age and heart failure, the top two predictive features (Supplemental Figure 2S). For those equal to or less than 70 years old, the SHAP value for the presence of heart failure was positive, but approximately zero for its absence, and vice versa for the age group above 70 years. However, the SHAP value for age, when excluding the interaction with other features (i.e., the main effect of age), showed slight dispersion with higher SHAP values associated with increasing age. Additionally, an interaction of length of stays and transfusion of blood/blood products was detected by SHAP analysis. Among patients who received a transfusion, those with length of stay of fewer than 14 days had positive SHAP value. In contrast, patients who did not receive a transfusion had a positive SHAP value when their length of stay exceeded 14 days. The adverse cutoff for age was >70 years (Supplemental Figure 2S). Minimal SHAP interaction values were found between other features.

### Hierarchical Clustering and Rules-Based Cluster Descriptions

**Figure 4.**
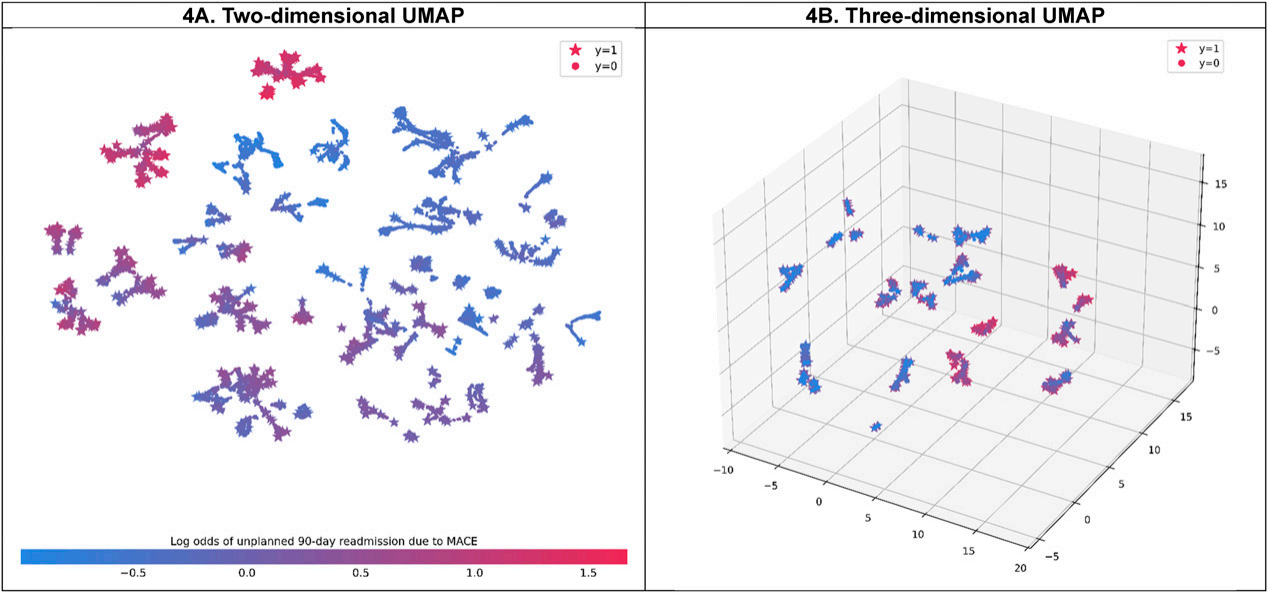
Uniform Manifold Approximation and Projection (UMAP) Embedding of SHapley Additive exPlanations (SHAP) Values by Dimension. Caption: The Left-Hand Plot Shows the Two-Dimensional UMAP Embedding of SHAP Values for the Whole Population (n = 76 957), while the Hand-Right Plot Shows Three-Dimensional UMAP Embedding of SHAP Values for the Readmitted and High-Risk Populations (n = 16 692).

Clustering based on a two-dimensional embedding of SHAP values, obtained from overall data and the CatBoost algorithm (which achieved the highest AUC values), showed distinct groups of patients (Figure 4 [left]). Identified clusters with higher log odds for unplanned 90-day MACE readmission were moderately separated from those with lower log odds for the outcome in general. Notably, several patients who experienced readmission were clustered in the lower log odds clusters, and several clusters were apart from each other despite having similar log odds for the outcome.

Clustering based on a three-dimensional embedding of SHAP values and rule-based descriptions for understanding the features that explain readmitted and high-risk patients (with log odds greater than the 75th percentile of the predicted log odds, 0.31) is shown in Figure 4 (right) and Supplemental Table 5. A total of 23 clusters were identified, with a maximum mean silhouette score of 0.752 achieved through fine-tuning of hyperparameters. In general, the description rules for clusters that achieved high precision scores ranged from 98.6% to 100.0%, while recall scores were acceptable, ranging from 59.6% to 100.0%. A cluster with the highest mean log odds (1.41 [95% CI, 1.39-1.41]), corresponding to 2.41% of the readmitted and high-risk population, was characterized by the presence of nonrheumatic and unspecified valve disorders, coronary atherosclerosis and other heart diseases, and heart failure. The second-largest mean log odds cluster consisted of patients who had coronary atherosclerosis and other heart diseases, heart failure, and type 2 diabetes, but without nonrheumatic and unspecified valve disorders.

## Discussion

We aimed to develop an ML algorithm for predicting the 90-day unplanned readmission due to MACE among hospitalized patients with blood cancers. Our ML models included a variety of demographics, admission/discharge details, and clinical variables, facilitating healthcare providers to predict the risk of unplanned readmission due to MACE. Additionally, we used a 90-day period to capture MACE readmissions. The narrower period (i.e., 30 days) could not fully capture the CV-related readmissions and elevated CVD risk,^33-36^ which patients with blood cancers are well-known for.^15,75^ We also used SHAP values and SHAP-based supervised clustering to improve model explainability, which can provide a readily interpretation and gain trust in clinical decision process.

By comparing six ML algorithms, we found that the predictive performance of those algorithms varied among classification metrics. On the held-out test set, CatBoost, LR-L2, and RF performed best when evaluating using AUCs (ROC and PR), $F_{2}$ score, and balanced Brier score, respectively. However, the differences in these metrics were not statistically significant between the aforementioned models and XGBoost. Talwar et al. conducted a meta-analysis comparing the performance of hospital readmission prediction models showed that although tree-based algorithms – decision tree, random forest, boosting – achieved higher AUC than logistic regression, the differences were not significant.^76^ In a simulation, Kirasich et al. showed that logistic regression performed with higher accuracy compared to random forest when increasing the variance of the explanatory and noise variables.^77^ This might be an explanation for the higher $F_{2}$ and balanced scores of logistic regression compared to tree-based algorithms in this study. Besides, the feature importance ranked by mean absolute SHAP values in our study (Supplemental Figure S3) showed the numbers of higher important features utilized by tree-based algorithms were smaller compared to LR-L2. A future study is warranted to explore changes in performance when varying the number of predictive features. Despite selecting a single algorithm for predicting, we utilized the SuperLearner algorithm to leverage the performance of these models. Several works on predicting readmission have shown that this method performed better than using only one single ML algorithm.^78-80^ As a result, our model also achieved a slight enhancement in performance compared to each single algorithm.

This study dealt with highly imbalanced data and cost-sensitive learning was considered to be appropriate for addressing this problem. Approaches for handling imbalanced data could be classified as preprocessing level (data level), such as Synthetic Minority Oversampling Technique (SMOTE) and learning level (algorithmic level), such as cost-sensitive learning.^81^ Studies have shown that these two approaches are comparable in dealing with imbalanced data.^82,83^ However, concerns regarding preprocessing approaches (e.g. oversampling, SMOTE) include the potential to produce data that may not precisely match the original distribution of the minority class, potentially impacting performance.^84-86^ Algorithmic-level approaches could address this issue; however, a significant limitation of cost-sensitive learning is the uncertainty of cost values.^59^ We addressed this limitation by considering the cost values as model hyperparameters and performing tuning to obtain the optimal cost setup for the models.^59^ Although this approach effectively addressed the imbalance problem, future research can continue to refine prediction models by testing different data imbalance solutions^47,87^ or utilizing additional ML algorithms to improve classification performance and provide a more comprehensive understanding of handling imbalanced data.

Previous readmission prediction models used traditional methods to explore the predictors of readmissions in patients with blood cancer.^18,20,21,88^ Kunapareddy et al used logistic regression to predict 30-day unplanned readmission in patients with blood malignancies. Their significant predictors included an absolute neutrophil count, fever, gastrointestinal symptoms, febrile neutropenia, and relapsed/refractory disease.^88^ Spring et al. also used logistic regression to determine the potential risk factors for readmissions after hematopoietic transplantation. Their predictors included infection during the index admission and active disease at the time of transplantation.^19^ While regression models are powerful prediction tools for readmission, they are typically limited to linear relationships and may not capture the interactions between the predictors.^89^ Especially in blood cancer, the complicated nature of treatments (e.g., chemotherapy, blood transfusions, and transplantations) and their associated side effects (e.g., infection and immunosuppression) make it especially difficult to predict readmissions in patients with blood cancer.^90^ Consequently, ML approaches can perform better by identifying subtle patterns and interactions that regression methods might neglect. This ultimately leads to improved prediction accuracy and more effective risk stratification for patients with blood cancers.^89^ Our ML models showed a new and higher number of significant predictors of readmission, providing a better prediction for readmissions in blood cancers.

We identified a range of predictors for 90-day unplanned MACE readmissions, which could be categorized into modifiable and non-modifiable factors. The categorization allows the providers to determine the appropriate care interventions.^91^ Modifiable factors include CV complications (HF, coronary atherosclerosis, cardiac dysrhythmia), cellular therapy procedures like administration and transfusion of bone marrow and stem cells, fever, neoplasm complications, and other comorbidities (diabetes and renal failure). Patients with blood cancers are exposed to several cardiotoxic cancer therapies, which might elevate the risk of developing subsequent CVD and increase the risk of unplanned hospital readmissions.^15,92-95^ Certain preventive strategies, including cardio-protective medications, regular monitoring, and chemotherapy dose adjustments, could be used to decrease the risk of CV-related unplanned readmissions.^96,97^ Additionally, neoplasm complications, including tumor lysis syndrome and infection, contribute to systemic inflammation and metabolic disturbances, leading to acute cardiac events. Managing neoplasm complications involves prompt treatment of infections and close management of metabolic abnormalities.^98^ Moreover, comorbidities like diabetes and renal failure worsen cardiovascular outcomes through mechanisms such as endothelial dysfunction and fluid overload. Appropriate control over these comorbidities is required to decrease the impact of these diseases on the CV system.^99^ Non-modifiable factors include older age, being a resident, routine discharge disposition, and length of stay. While non-modifiable factors such as age and residency status cannot be altered, their impact on readmissions can be mitigated through tailored strategies.

The SHAP values and SHAP-based clustering analyses provided insights into addressing clinicians’ understandable hesitancy toward incorporating ML outputs into clinical decision-making. The SHAP analysis (Figure 2S) showed that patients who aged >70 years old had positive SHAP values, indicating they experienced higher 90-day unplanned MACE readmissions compared to overall population. The SHAP analysis also indicated an interaction between age and heart failure, with the presence of heart failure in patients aged >70 years old having negative SHAP values compared to those aged ≤70 years old. Although this effect was negligible compared to the main effect of age, this counterintuitive association might be due to competing risks, such as non-CVD mortality, that prevent observations of unplanned MACE outcomes among older patients. Further studies are warranted to explore this, and interpreting the results should be done cautiously. Moreover, we also found the interaction between length of stay and transfusion of blood/blood products in SHAP analysis. Blood transfusion is generally associated with increased hospital length of stay because of the associated complications and the need for longer monitoring.^100^ Patients with blood transfusion and a short length of stay may have been discharged while still being at risk of complications (e.g. anemia, infection, and hemodynamic instability), increasing their admission risk compared to patients with prolonged length of stay. Appropriate discharge planning and close monitoring are likely needed in patients with blood transfusion and short length of stay to decrease the risk of MACE readmission. These results suggest that strategies, including comprehensive geriatric assessments, early mobilization, and appropriate discharge planning for these patients may prevent unplanned readmissions.

Moreover, SHAP-based clustering with decision rules helps identify high-risk patients for MACE readmissions using binary decision on selected variables. For example, by determining the presence of heart failure, nonrheumatic and unspecified valve disorders, coronary atherosclerosis and other heart disease, clinicians can rapidly classify patients as high-risk for MACE readmissions. Therefore, early preventive interventions within hospitalization and discharge management strategies could be implemented to prevent readmissions.^101^ These interventions may include scheduling an outpatient follow-up visit within seven to 10 days of hospital discharge , an approach shown to reduce unplanned readmissions in cancer patients and lower the financial burden on healthcare systems.^91,102^ Additional interventions could include improving patient access to medical services, standardizing clinical and symptom management, and continuous patient monitoring.^103^

Our study had several limitations that should be addressed. First, due to the nature of NRD database, potential predictive features of hospital readmissions are not available, for instance, chemotherapy regimen details, cancer stages, and laboratory test results. Therefore, we could not evaluate the impact of these features on prediction performance. In addition to these factors, race/ethnicity is unavailable, limiting us from evaluating the fairness of our models. However, including socioeconomic status (SES) variables—such as urban-rural location, health insurance, and residency status—in our models may help mitigate fairness biases across patient groups. Race and SES are closely linked, with studies showing that SES factors mediate a significant portion of racial disparities in cardiovascular outcomes among patients with cancer^104^ and that incorporating social determinants of health can improve fairness while preserving predictive accuracy.^105^ We also acknowledged the heterogeneity in the definitions of MACE. While our study defined MACE outcomes based on a thorough review by Bosco et al,^43^ some events might not have been fully captured. To address this, we reported the administrative codes used to mitigate potential of misleading interpretations of the results. Malignant arrhythmias, including ventricular tachycardia (VT), ventricular fibrillation (VF), and torsades de pointes, are major outcomes in patients with blood cancer. According to Enriquez et al, almost one-third of patients with an implantable cardioverter-defibrillator developed VT after a cancer diagnosis, including those with blood cancer.^106^ Multiple factors, such as electrolyte imbalance, the arrhythmogenic effects of medications, and cardiotoxicity, are among the leading causes of malignant arrhythmias.^107^ However, only nine cases of malignant arrhythmias were identified, and among these, eight patients died and were classified as any-CVD-cause mortality. Given their clinical significance, we suggest including malignant arrhythmias in the MACE definition in future studies. Additionally, studies that include a larger sample of malignant arrhythmia cases could better assess their impact on readmission risk and provide a more comprehensive perspective on patient outcomes and treatment efficacy. Moreover, we could not assess the generalizability of our models when applying external data, such as local electronic health records. Future efforts can be focused on including more and selecting important predictors to improve the performance of models, evaluating fairness metrics to ensure fair predictions among groups, and validating using external data. The advanced performance may be affected by the highly imbalanced data – the small number of MACE readmission events. Due to the nature of the NRD HCUP data, patients discharged in the last quarter of each year were excluded because our primary outcome was a 90-day unplanned MACE hospital readmission. Therefore, interpreting the results for patients discharged in the last quarter should be carried out with caution. Finally, while we primarily focused on MACE-related readmissions, a broader objective to explore non-MACE related readmissions, such as those due to neutropenia, sepsis, etc. is warranted to provide a more comprehensive perspective on readmissions among patients with blood cancer.

## Conclusion

Our study presented the applicability and performance of ML approaches in predicting the risk of MACE-related readmission in patients with blood cancers. The study identified a range of modifiable predictors, such as CV complications (HF, coronary atherosclerosis, cardiac dysrhythmia) and other comorbidities (diabetes and renal failure) and non-modifiable predictors, such as older age and length of stay, for unplanned MACE readmission that can be used to develop appropriate discharge care strategies. Putting these strategies into action can substantially affect healthcare systems, as they can potentially decrease the burden of blood cancers and lower the overall costs associated with readmissions.

## Supplemental Material

Supplemental material - Machine Learning-Based Prediction of Unplanned Readmission Due to Major Adverse Cardiac Events Among Hospitalized Patients with Blood Cancers Supplemental Material for Machine Learning-Based Prediction of Unplanned Readmission Due to Major Adverse Cardiac Events Among Hospitalized Patients with Blood Cancers by Nguyen Le, Sola Han, Ahmed S. Kenawy, Yeijin Kim, and Chanhyun Park in Cancer Control

## ORCID iDs

Nguyen Le https://orcid.org/0000-0002-4233-4696

Sola Han https://orcid.org/0000-0002-6269-172X

Ahmed S. Kenawy https://orcid.org/0000-0002-5133-265X

Yeijin Kim https://orcid.org/0000-0002-3183-8477

Chanhyun Park https://orcid.org/0000-0002-1081-0950

## Statements and Declarations

### Ethical Approval

This study used publicly available deidentified data obtained from the Nationwide Readmissions Database. The Institutional Review Board of The University of Texas at Austin exempted the study, and informed consent was not required.
