# Supplementary material for: Machine Learning-Based Prediction of Unplanned Readmission Due to Major Adverse Cardiac Events Among Hospitalized Patients with Blood Cancers
Source: Cancer Control. 2025 Apr 17;32:10732748251332803. doi: 10.1177/10732748251332803 (PMC12035306; doi:10.1177/10732748251332803)

SUPPLEMENTARY MATERIAL

Table S1. Codes were used in the study 1
Table S2. The list of features included in training and validating models 2
Table S3. Hyperparameters tuning range for the candidate machine learning models............... 5
Table S4. Statistical testing for evaluating classification performance metrics 6
Table S5. Subgroup identification for readmitted and high-risk patients (n= 19619).................... 7
Figure 1S. Feature importance (SHAP values bar plots) of developed models..........................10
Figure 2S. Partial dependence plots of Age (A) and Length of stay (B) 14
Figure 3S. Predicted log odds quantiles for the study population 16

Table S1. Codes were used in the study

Diagnosis/Procedure	Codes (HCUP Clinical Classifications Software Refined)
**Blood cancers**	
Leukemia	NEO059, NEO060, NEO061, NEO062, NEO063, NEO064
Lymphoma	NEO057, NEO058
Multiple myeloma	NEO065
Myelodysplastic syndrome (MDS)	NEO068
**MACE**	
Acute coronary syndrome	CIR011
Acute myocardial infarction	CIR009, CIR010
Heart failure	CIR019
Myocarditis and cardiomyopathy	CIR005
Arrhythmia	CIR019, only included I462, I468, I469, I4901, I4902, I97120, I97121, I97710, I97711 (ICD-10-CM diagnoses)
Stroke and transient ischemic attack	CIR020, CIR021, CIR023, CIR024, NVS012
Cardiovascular mortality	Patients got primary diagnosis with CIR*** and died during readmitted hospitalization
Revascularization (coronary artery bypass grafting, percutaneous coronary intervention)	0210, 0211, 0212, 0213, 0270, 0271, 0272, 0273 (ICD-10-PCS procedures)
HCUP Clinical Classifications Software Refined (CCSR) for ICD-10-CM Diagnoses	We used the v2023.1 version can be found at Clinical Classifications Software Refined (CCSR) for ICD-10-CM Diagnoses (ahrq.gov)
HCUP Clinical Classifications Software Refined (CCSR) for ICD-10-PCS Procedures	We used the v2023.1 version can be found at Clinical Classifications Software Refined (CCSR) for ICD-10-PCS Procedures (ahrq.gov)

Table S2. The list of features included in training and validating models

**DEMOGRAPHICS**	
Age (years)	Sex (Female)
Resident	Medicare, Medicaid, Private, Other (Seft-pay, No charge, Other) Insurance (reference: Medicare)
Urban-Rural location (reference: "Central" counties >=1 million population)	Household income (reference: Quartile 4)
**ADMISSION/DISCHARGE**	
Discharge month (reference: February)	Weekend admission
Discharge quarter (reference: Quarter 1)	Length of stay (days)
Discharge disposition (Routine, Transfer to Short-term Hospital, Skilled Nursing/Intermediate Care Facility, Home Health Care) (reference: Routine)	
**HCUP CLINICAL CLASSIFICATIONS SOFTWARE REFINED FOR DIAGNOSES**	
Nutritional anemia (BLD001)	Other specified and unspecified diseases of kidney and ureters (GEN006)
Hemolytic anemia (BLD002)	Other specified and unspecified diseases of bladder and urethra (GEN007)
Aplastic anemia (BLD003)	Urinary incontinence (GEN008)
Acute posthemorrhagic anemia (BLD004)	Hematuria (GEN009)
Coagulation and hemorrhagic disorders (BLD006)	Hyperplasia of prostate (GEN012)
Diseases of white blood cells (BLD007)	Other specified male genital disorders (GEN016)
Immunity disorders (BLD008)	Septicemia (INF002)
Other specified and unspecified hematologic conditions (BLD010)	Bacterial infections (INF003)
Chronic rheumatic heart disease (CIR001)	Fungal infections (INF004)
Nonrheumatic and unspecified valve disorders (CIR003)	Hepatitis (INF007)
Myocarditis and cardiomyopathy (CIR005)	Viral infection (INF008)
Pericarditis and pericardial disease (CIR006)	Superficial injury; contusion, initial encounter (INJ017)
Essential hypertension (CIR007)	Adverse effects of drugs and medicaments, initial encounter (INJ028)
Hypertension with complications and secondary hypertension (CIR008)	Drug induced or toxic related condition (INJ030)
Acute myocardial infarction (CIR009)	Allergic reactions (INJ031)
Coronary atherosclerosis and other heart disease (CIR011)	Complication of cardiovascular device, implant or graft, initial encounter (INJ033)
Nonspecific chest pain (CIR012)	Complication of transplanted organs or tissue, initial encounter (INJ036)
Acute pulmonary embolism (CIR013)	Complication of other surgical or medical care, injury, initial encounter (INJ037)
Pulmonary heart disease (CIR014)	Depressive disorders (MBD002)
Conduction disorders (CIR016)	Bipolar and related disorders (MBD003)
Cardiac dysrhythmias (CIR017)	Anxiety and fear-related disorders (MBD005)
Heart failure (CIR019)	Alcohol-related disorders (MBD017)
Cerebral infarction (CIR020)	Opioid-related disorders (MBD018)
Acute hemorrhagic cerebrovascular disease (CIR021)	Cannabis-related disorders (MBD019)
Occlusion or stenosis of precerebral or cerebral arteries without infarction (CIR023)	Tobacco-related disorders (MBD024)
Sequela of cerebral infarction and other cerebrovascular disease (CIR025)	Rheumatoid arthritis and related disease (MUS003)
Peripheral and visceral vascular disease (CIR026)	Osteoarthritis (MUS006)
Aortic; peripheral; and visceral artery aneurysms (CIR029)	Musculoskeletal pain, not low back pain (MUS010)
Hypotension (CIR031)	Spondylopathies/spondyloarthropathy (including infective) (MUS011)
Other specified and unspecified circulatory disease (CIR032)	Osteoporosis (MUS013)
Acute phlebitis; thrombophlebitis and thromboembolism (CIR033)	Pathological fracture, initial encounter (MUS014)
Postthrombotic syndrome and venous insufficiency/hypertension (CIR036)	Stress fracture, initial encounter (MUS016)
Other specified diseases of veins and lymphatics (CIR039)	Systemic lupus erythematosus and connective tissue disorders (MUS024)
Any dental condition including traumatic injury (DEN001)	Other specified connective tissue disease (MUS025)
Nontraumatic dental conditions (DEN002)	Muscle disorders (MUS026)
Caries, periodontitis, and other preventable dental conditions (DEN003)	Other specified bone disease and musculoskeletal deformities (MUS028)
Intestinal infection (DIG001)	Gout (MUS033)
Disorders of teeth and gingiva (DIG002)	Low back pain (MUS038)
Diseases of mouth; excluding dental (DIG003)	Male reproductive system cancers - prostate (NEO039)
Esophageal disorders (DIG004)	Non-Hodgkin lymphoma (NEO058)

Gastroduodenal ulcer (DIG005)	Leukemia - acute myeloid leukemia (AML) (NEO060)
Gastritis and duodenitis (DIG007)	Leukemia - chronic myeloid leukemia (CML) (NEO062)
Other specified and unspecified disorders of stomach and duodenum (DIG008)	Myelodysplastic syndrome (MDS) (NEO068)
Abdominal hernia (DIG010)	Secondary malignancies (NEO070)
Intestinal obstruction and ileus (DIG012)	Neoplasms of unspecified nature or uncertain behavior (NEO072)
Diverticulosis and diverticulitis (DIG013)	Benign neoplasms (NEO073)
Hemorrhoids (DIG014)	Conditions due to neoplasm or the treatment of neoplasm (NEO074)
Anal and rectal conditions (DIG015)	Other nervous system disorders (often hereditary or degenerative) (NVS006)
Biliary tract disease (DIG017)	Paralysis (other than cerebral palsy) (NVS008)
Other specified and unspecified liver disease (DIG019)	Epilepsy; convulsions (NVS009)
Gastrointestinal hemorrhage (DIG021)	Headache; including migraine (NVS010)
Noninfectious gastroenteritis (DIG022)	Neurocognitive disorders (NVS011)
Other specified and unspecified gastrointestinal disorders (DIG025)	Polyneuropathies (NVS015)
Hearing loss (EAR004)	Sleep wake disorders (NVS016)
Thyroid disorders (END001)	Nerve and nerve root disorders (NVS017)
Diabetes mellitus without complication (END002)	Nervous system pain and pain syndromes (NVS019)
Diabetes mellitus with complication (END003)	Other nervous system disorders (neither hereditary nor degenerative) (NVS020)
Diabetes mellitus, Type 2 (END005)	Pneumonia (except that caused by tuberculosis) (RSP002)
Nutritional deficiencies (END007)	Acute bronchitis (RSP005)
Malnutrition (END008)	Other specified upper respiratory infections (RSP006)
Obesity (END009)	Other specified and unspecified upper respiratory disease (RSP007)
Disorders of lipid metabolism (END010)	Chronic obstructive pulmonary disease and bronchiectasis (RSP008)
Fluid and electrolyte disorders (END011)	Asthma (RSP009)
Pituitary disorders (END013)	Aspiration pneumonitis (RSP010)
Other specified and unspecified endocrine disorders (END015)	Pleurisy, pleural effusion and pulmonary collapse (RSP011)
Other specified and unspecified nutritional and metabolic disorders (END016)	Respiratory failure; insufficiency; arrest (RSP012)
External cause codes: fall; initial encounter (EXT003)	Other specified and unspecified lower respiratory disease (RSP016)
External cause codes: intent of injury, accidental/unintentional (EXT020)	Skin and subcutaneous tissue infections (SKN001)
External cause codes: complications of medical and surgical care, initial encounter (EXT025)	Other specified inflammatory condition of skin (SKN002)
External cause codes: place of occurrence of the external cause (EXT027)	Pressure ulcer of skin (SKN003)
Cataract and other lens disorders (EYE002)	Non-pressure ulcer of skin (SKN004)
Glaucoma (EYE003)	Postprocedural or postoperative skin complication (SKN006)
Retinal and vitreous conditions (EYE005)	Other specified and unspecified skin disorders (SKN007)
Blindness and vision defects (EYE010)	Syncope (SYM001)
Implant, device or graft related encounter (FAC009)	Fever (SYM002)
Other aftercare encounter (FAC010)	Shock (SYM003)
Medical examination/evaluation (FAC014)	Nausea and vomiting (SYM004)
Exposure, encounters, screening or contact with infectious disease (FAC016)	Dysphagia (SYM005)
Socioeconomic/psychosocial factors (FAC019)	Abdominal pain and other digestive/abdomen signs and symptoms (SYM006)
Personal/family history of disease (FAC021)	Malaise and fatigue (SYM007)
Acquired absence of limb or organ (FAC022)	Nervous system signs and symptoms (SYM010)
Organ transplant status (FAC023)	Genitourinary signs and symptoms (SYM011)
Other specified status (FAC025)	Circulatory signs and symptoms (SYM012)
Nephritis; nephrosis; renal sclerosis (GEN001)	Respiratory signs and symptoms (SYM013)
Acute and unspecified renal failure (GEN002)	Skin/Subcutaneous signs and symptoms (SYM014)
Chronic kidney disease (GEN003)	General sensation/perception signs and symptoms (SYM015)
Urinary tract infections (GEN004)	Other general signs and symptoms (SYM016)
Calculus of urinary tract (GEN005)	Abnormal findings without diagnosis (SYM017)
**HCUP CLINICAL CLASSIFICATIONS SOFTWARE REFINED FOR PROCEDURES**	

Transfusion of blood and blood products (ADM001)	Paracentesis (GIS006)
Transfusion of plasma (ADM002)	Chest wall procedures, NEC (GNR006)
Infusion of vasopressor (ADM006)	Retroperitoneal procedures, NEC (GNR009)
Administration and transfusion of bone marrow, stem cells, pancreatic islet cells, and t-cells (ADM011)	Cardiac and coronary fluoroscopy (IMG001)
Chemotherapy (ADM012)	Fluoroscopic angiography (excluding coronary) (IMG002)
Administration of anti-inflammatory agents (ADM016)	Fluoroscopic guidance for circulatory system procedures (IMG003)
Administration of nutritional and electrolytic substances (ADM017)	Fluoroscopy of non-circulatory organs (IMG004)
Administration of therapeutic substances, NEC (ADM021)	Computerized tomography (CT) with contrast (IMG006)
Venous and arterial catheter placement (CAR024)	Ultrasonography (IMG008)
Placement of tunneled or implantable portion of a vascular access device (CAR025)	Lymph node biopsy (LYM001)
Cardiovascular device procedures, NEC (CAR029)	Bone marrow biopsy (LYM007)
Minimally invasive CNS biopsy (CNS001)	Measurement during cardiac catheterization (MAM005)
Lumbar puncture (CNS002)	Bone and joint biopsy (MST002)
CNS excision procedures (CNS007)	Subcutaneous tissue, fascia, and muscle biopsy (MST003)
Laryngoscopy (diagnostic) (ENT001)	Beam radiation (RAD001)
ENT diagnostic procedures (non-endoscopic) (ENT003)	Bronchoscopy (diagnostic) (RES001)
Hemodialysis (ESA001)	Thoracentesis (diagnostic) (RES003)
Mechanical ventilation (ESA003)	Chest tube placement and therapeutic thoracentesis (RES005)
Non-invasive ventilation (ESA004)	Airway intubation (RES007)
Pheresis therapy (EST001)	Skin biopsy and diagnostic drainage (SKB001)
Esophagogastroduodenoscopy (EGD) with biopsy (GIS001)	Kidney and other urinary tract biopsy (non-endoscopic) (URN002)
Colonoscopy and proctoscopy with biopsy (GIS002)	Ureter and other urinary tract dilation (URN003)

Table S3. Hyperparameters tuning range for the candidate machine learning models

ML algorithm	Hyperparameter	Search range	Best value
Logistic regression with L2 regularization	C (inverse of regularization strength)	[0.00001, 100000]	133.8
	class_weight	[39, 117]	39
	penalty = “l2”, solver = “lbfgs”		
Support Vector Machine	kernal	[“linear”, “poly”, “rbf”, “sigmoid”]	“sigmoid”
	C (inverse of regularization strength)	[0.00001, 100000]	53.2
	gamma	[0.00001, 100000]	2.27×10^{-5}
	class_weight	[39, 117]	39
Complement Naïve Bayes	alpha	[0.00001, 100000]	15.6
	fit_prior	[False, True]	True
Random Forest	n_estimators	[100, 10000]	9383
	criterion	[“gini”, “entropy”, “log_loss”]	“log_loss”
	min_samples_split	[2, 100]	67
	min_samples_leaf	[1, 10]	6
	min_weight_fraction_leaf	[0.0, 0.5]	0.005
	max_features	[“sqrt”, “log2”]	“sqrt”
	class_weight	[39, 117]	65.4
XGBoost	learning_rate	[0.00001, 1]	0.007
	n_estimators	[200, 1000]	623
	gamma	[1, 9]	8
	max_depth	[3, 18]	17
	reg_alpha	[0.00001, 100000]	0.943
	reg_lambda	[0.00001, 100000]	303.13
	colsample_bytree	[0.5, 1]	0.888
	min_child_weight	[0.001, 1000]	8.3
	class_weight	[39, 117]	72.51
CatBoost	learning_rate	[0.00001, 1]	0.003
	n_estimators	[200, 1000]	927
	od_pval	[10^{-10}, 0.001]	4.3×10^{-9}
	max_depth	[3, 18]	4
	l2_leaf_reg	[0.00001, 100000]	2897.31
	bagging_temperature	[0, 10]	1.5
	min_child_sample	[2, 100]	39
	class_weight	[39, 117]	45.0
	grow_policy = “Depthwise”		
Super learner	class_weight	[39, 117]	39
HDBSCAN	min_cluster_size	[11, 200]	86
	min_samples	[11, 200]	200
	cluster_selection_epsilon	[0, 8]	1.0006

Table S4. Statistical testing for evaluating classification performance metrics

**ROC-AUC – Delong's test**						
	**LR**	**SVM**	**CNB**	**RF**	**XGB**	**CB**
**LR**	-					
**SVM**	0.0049	-				
**CNB**	**<0.0001**	**<0.0001**	-			
**RF**	0.7195	0.0173	**<0.0001**	-		
**XGB**	0.1131	**<0.0001**	**<0.0001**	0.1326	-	
**CB**	0.0119	**<0.0001**	**<0.0001**	0.0161	0.2647	**-**
**PR-AUC – Permutation test**						
	**LR**	**SVM**	**CNB**	**RF**	**XGB**	**CB**
**LR**	-					
**SVM**	0.935	-				
**CNB**	**<0.0001**	**<0.0001**	-			
**RF**	0.788	0.797	**<0.0001**	-		
**XGB**	0.636	0.864	**<0.0001**	0.385	-	
**CB**	0.633	0.895	**<0.0001**	0.368	0.915	**-**
**F2 – Friedman post-hoc test with Finner's correction**						
	**LR**	**SVM**	**CNB**	**RF**	**XGB**	**CB**
**LR**	-					
**SVM**	0.1312	-				
**CNB**	**0.0020**	0.1312	-			
**RF**	0.9592	0.1312	**0.0020**	-		
**XGB**	0.6956	0.2563	**0.0049**	0.6954	-	
**CB**	0.9401	0.1312	**0.0020**	0.9200	0.7258	**-**
**Balanced Brier score – correlated t-test**						
	**LR**	**SVM**	**CNB**	**RF**	**XGB**	**CB**
**LR**	-					
**SVM**	**<0.0001**	-				
**CNB**	<0.0001	**<0.0001**	-			
**RF**	0.9349	**<0.0001**	**<0.0001**	-		
**XGB**	0.9074	**<0.0001**	**<0.0001**	0.3600	-	
**CB**	0.9740	**<0.0001**	**<0.0001**	0.9990	0.47533	**-**

Bold values represent statistically significant results at the $p < 0.0033$ (Bonferroni-corrected with 15 comparisons) for the Delong's, Permutation and corrected tests; the $p < 0.05$ for the Friedman post-hoc test.

Table S5. Subgroup identification for readmitted and high-risk patients (n= 16692)

Cluster	N (%)	Readmitted rate	Mean log odd (95% CI)	Rules	Precision	Recall
0	1347 (8.07%)	35 (2.60%)	0.82 (0.81-0.83)	Nonrheumatic and unspecified valve disorders (No) and Administration and transfusion of bone marrow, stem cells, pancreatic islet cells, and t-cells (No) and Coronary atherosclerosis and other heart disease (No) and Cardiac dysrhythmias (No) and Heart failure (Yes) and Diabetes mellitus, Type 2 (No)	100.0%	100.0%
1	579 (3.47%)	16 (2.76%)	0.37 (0.37-0.37)	Age > 71.5 and Nonrheumatic and unspecified valve disorders (No) and Hypertension with complications and secondary hypertension (No) and Coronary atherosclerosis and other heart disease (No) and Cardiac dysrhythmias (No) and Heart failure (No) and Diabetes mellitus without complication (Yes) and Implant, device or graft related encounter (No) and Acute and unspecified renal failure (No)	100.0%	69.3%
2	277 (1.66%)	<11	0.4 (0.39-0.4)	Nonrheumatic and unspecified valve disorders (No) and Hypertension with complications and secondary hypertension (No) and Coronary atherosclerosis and other heart disease (No) and Cardiac dysrhythmias (No) and Heart failure (No) and Disorders of teeth and gingiva (No) and Diabetes mellitus without complication (Yes) and Implant, device or graft related encounter (No) and Other aftercare encounter (No) and Acute and unspecified renal failure (Yes)	98.5%	56.4%
3	957 (5.73%)	32 (3.34%)	0.45 (0.44-0.46)	Age > 66.0 and Nonrheumatic and unspecified valve disorders (No) and Hypertension with complications and secondary hypertension (Yes) and Coronary atherosclerosis and other heart disease (No) and Cardiac dysrhythmias (No) and Heart failure (No) and Diabetes mellitus, Type 2 (Yes)	100.0%	76.9%
4	530 (3.18%)	23 (4.34%)	0.55 (0.54-0.56)	Age > 58.5 and Nonrheumatic and unspecified valve disorders (Yes) and Coronary atherosclerosis and other heart disease (No) and Heart failure (No)	99.8%	99.8%
5	539 (3.23%)	16 (2.97%)	0.52 (0.51-0.53)	Age > 62.5 and Nonrheumatic and unspecified valve disorders (No) and Coronary atherosclerosis and other heart disease (No) and Cardiac dysrhythmias (Yes) and Heart failure (No) and Diabetes mellitus, Type 2 (Yes)	100.0%	100.0%
6	1017 (6.09%)	40 (3.93%)	1.04 (1.03-1.05)	Nonrheumatic and unspecified valve disorders (No) and Coronary atherosclerosis and other heart disease (No) and Heart failure (Yes) and Diabetes mellitus, Type 2 (Yes)	100.0%	100.0%
7	357 (2.14%)	11 (3.08%)	0.47 (0.46-0.48)	Age > 64.5 and Nonrheumatic and unspecified valve disorders (No) and Coronary atherosclerosis and other heart disease (No) and Cardiac dysrhythmias (Yes) and Heart failure (No) and Diabetes mellitus, Type 2 (No) and Implant, device or graft related encounter (Yes)	100.0%	92.8%
8	933 (5.59%)	33 (3.54%)	0.42 (0.41-0.42)	Age > 68.0 and Nonrheumatic and unspecified valve disorders (No) and Coronary atherosclerosis and other heart disease (No) and Cardiac dysrhythmias (Yes) and Heart failure (No) and Diabetes mellitus, Type 2 (No) and Implant, device or graft related encounter (No) and Other aftercare encounter (No)	100.0%	100.0%
9	373 (2.23%)	26 (6.97%)	1.19 (1.17-1.21)	Nonrheumatic and unspecified valve disorders (Yes) and Coronary atherosclerosis and other heart disease (No) and Heart failure (Yes)	100.0%	100.0%
10	1063 (6.37%)	33 (3.10%)	0.9 (0.89-0.92)	Nonrheumatic and unspecified valve disorders (No) and Coronary atherosclerosis and other heart disease (No) and Cardiac dysrhythmias	100.0%	86.5%

				(Yes) and Heart failure (Yes) and Diabetes mellitus, Type 2 (No)		
11	403 (2.41%)	26 (6.45%)	1.4 (1.39-1.41)	Nonrheumatic and unspecified valve disorders (Yes) and Coronary atherosclerosis and other heart disease (Yes) and Heart failure (Yes)	100.0%	100.0%
12	1172 (7.02%)	39 (3.33%)	0.56 (0.55-0.57)	Nonrheumatic and unspecified valve disorders (No) and Coronary atherosclerosis and other heart disease (Yes) and Cardiac dysrhythmias (No) and Heart failure (No) and Diabetes mellitus, Type 2 (No) and Implant, device or graft related encounter (Yes)	100.0%	93.6%
13	761 (4.56%)	28 (3.68%)	0.51 (0.5-0.51)	Age > 66.5 and Nonrheumatic and unspecified valve disorders (No) and Coronary atherosclerosis and other heart disease (Yes) and Cardiac dysrhythmias (No) and Heart failure (No) and Diabetes mellitus, Type 2 (No) and Implant, device or graft related encounter (No) and Other aftercare encounter (No)	100.0%	100.0%
14	1136 (6.81%)	44 (3.87%)	0.71 (0.7-0.71)	Nonrheumatic and unspecified valve disorders (No) and Coronary atherosclerosis and other heart disease (Yes) and Cardiac dysrhythmias (Yes) and Heart failure (No)	100.0%	100.0%
15	181 (1.08%)	<11	0.35 (0.35-0.36)	Age > 75.5 and Nonrheumatic and unspecified valve disorders (No) and Coronary atherosclerosis and other heart disease (No) and Cardiac dysrhythmias (No) and Heart failure (No) and Diabetes mellitus, Type 2 (No) and Implant, device or graft related encounter (No) and Acute and unspecified renal failure (No) and Chronic kidney disease (Yes)	98.6%	98.6%
16	903 (5.41%)	50 (5.53%)	1.27 (1.26-1.28)	Nonrheumatic and unspecified valve disorders (No) and Coronary atherosclerosis and other heart disease (Yes) and Heart failure (Yes) and Diabetes mellitus, Type 2 (Yes)	100.0%	100.0%
17	1250 (7.49%)	41 (3.28%)	0.64 (0.63-0.64)	Nonrheumatic and unspecified valve disorders (No) and Coronary atherosclerosis and other heart disease (Yes) and Cardiac dysrhythmias (No) and Heart failure (No) and Diabetes mellitus, Type 2 (Yes)	100.0%	100.0%
18	480 (2.88%)	26 (5.42%)	0.81 (0.8-0.83)	Nonrheumatic and unspecified valve disorders (Yes) and Coronary atherosclerosis and other heart disease (Yes) and Heart failure (No)	100.0%	100.0%
19	417 (2.50%)	17 (4.08%)	0.37 (0.37-0.38)	Age > 66.5 and Nonrheumatic and unspecified valve disorders (No) and Coronary atherosclerosis and other heart disease (No) and Cardiac dysrhythmias (No) and Heart failure (No) and Diabetes mellitus, Type 2 (No) and Implant, device or graft related encounter (Yes)	100.0%	100.0%
20	807 (4.83%)	35 (4.34%)	1.07 (1.06-1.08)	Nonrheumatic and unspecified valve disorders (No) and Coronary atherosclerosis and other heart disease (Yes) and Cardiac dysrhythmias (No) and Heart failure (Yes) and Diabetes mellitus, Type 2 (No)	100.0%	90.5%
21	459 (2.75%)	21 (4.58%)	1.24 (1.23-1.25)	Age > 67.5 and Nonrheumatic and unspecified valve disorders (No) and Coronary atherosclerosis and other heart disease (Yes) and Cardiac dysrhythmias (Yes) and Heart failure (Yes) and Anal and rectal conditions (No) and Diabetes mellitus, Type 2 (No) and Medical examination/evaluation (No)	99.9%	99.7%
22	361 (2.16%)	15 (4.16%)	0.38 (0.38-0.39)	Age > 70.0 and Nonrheumatic and unspecified valve disorders (No) and Hypertension with complications and secondary hypertension (Yes) and Coronary atherosclerosis and other heart disease (No) and Cardiac dysrhythmias (No) and Heart failure (No) and Diabetes mellitus, Type 2 (No) and Implant, device or graft related encounter (No) and Acute and unspecified renal failure (Yes)	100.0%	100.0%

23	390 (2.34%)	297 (76.15%)	-0.02 (-0.05-0.01)	Age > 59.5 and Nonrheumatic and unspecified valve disorders (No) and Hypertension with complications and secondary hypertension (No) and Coronary atherosclerosis and other heart disease (No) and Cardiac dysrhythmias (No) and Heart failure (No) and Diabetes mellitus, Type 2 (No) and Implant, device or graft related encounter (No)	100.0%	59.6%

Figure 1S. Feature importance (SHAP values bar plots) of developed models.

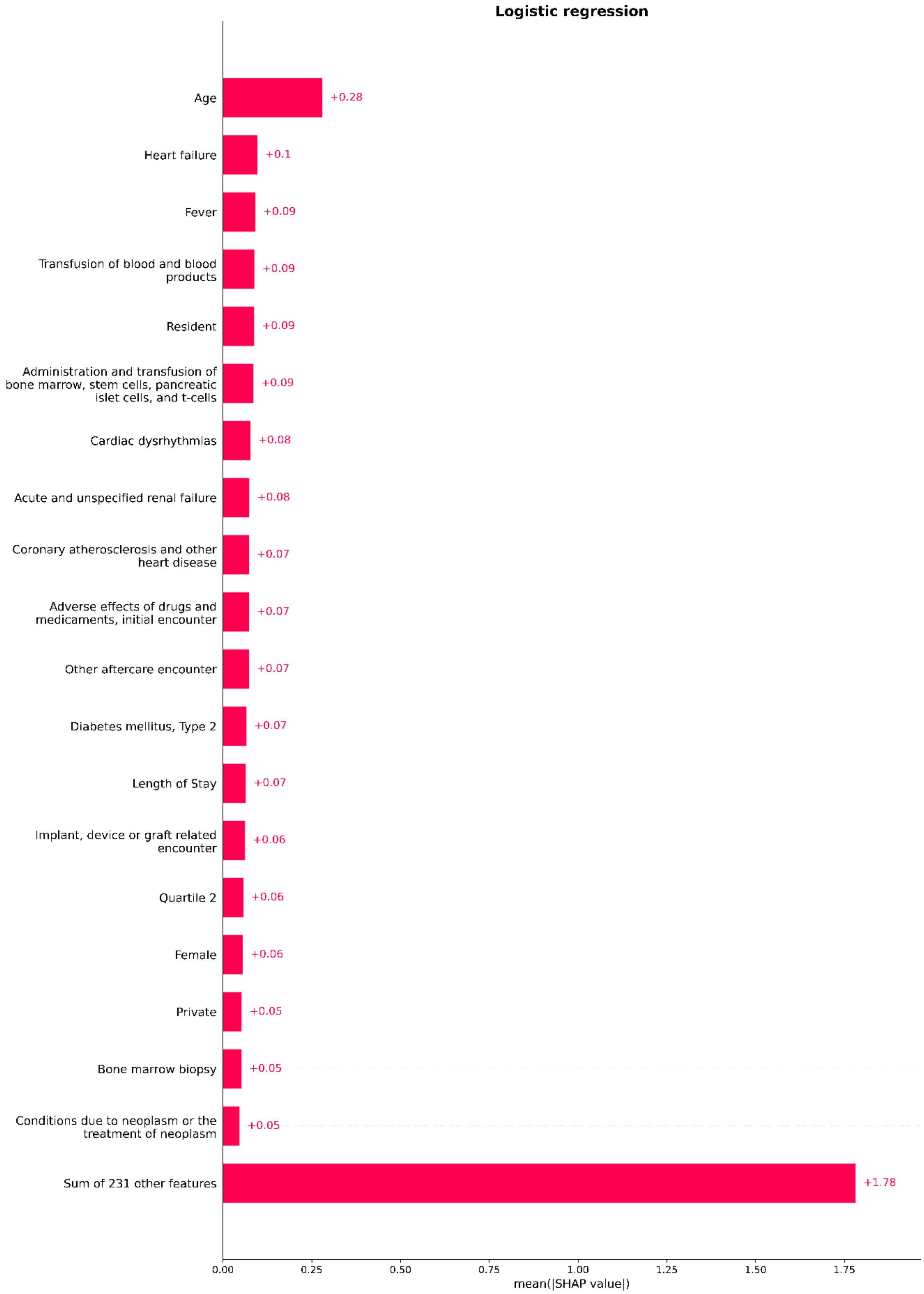

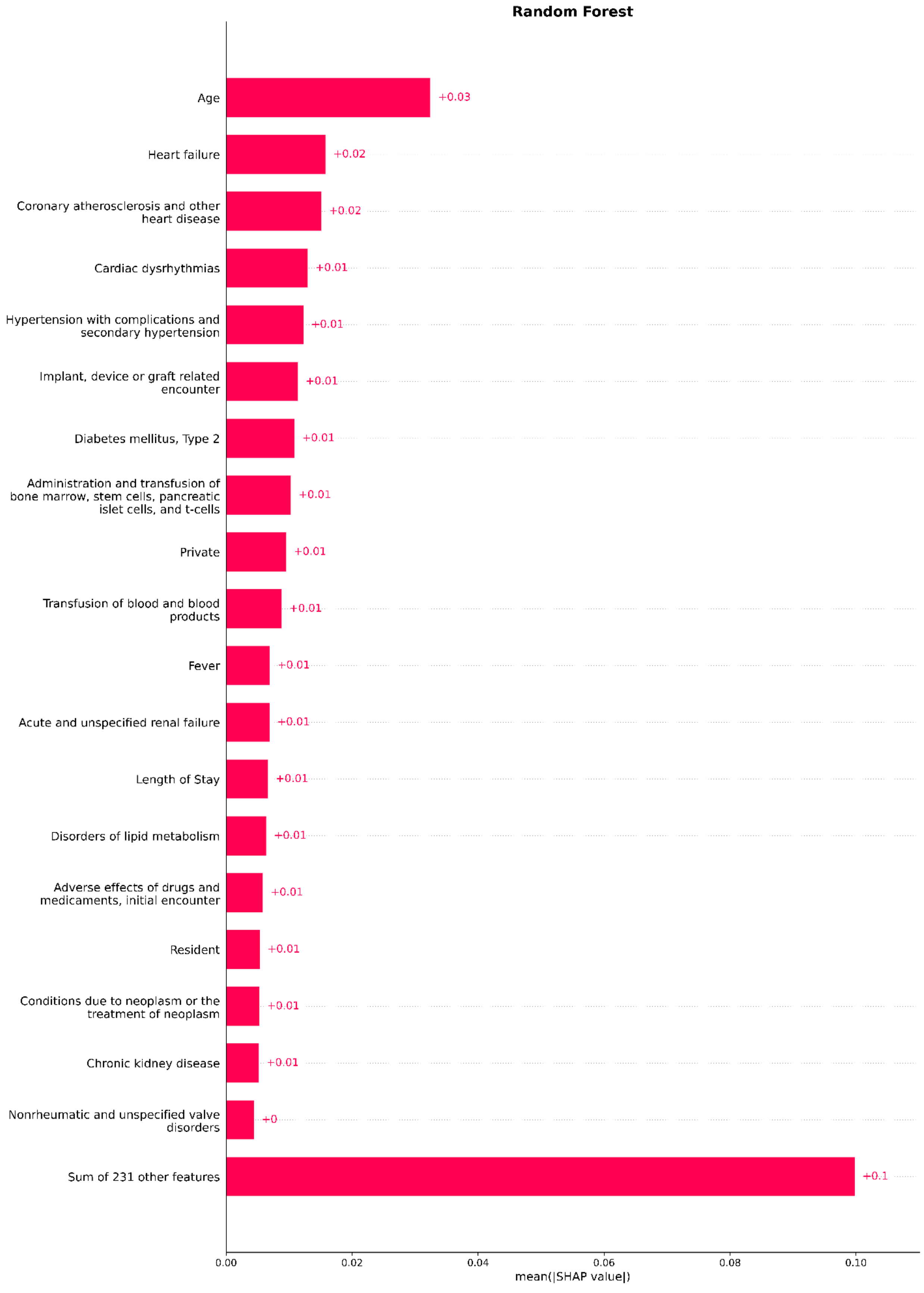
Random Forest
Age
+0.03
Heart failure
+0.02
Coronary atherosclerosis and other heart disease
+0.02
Cardiac dysrhythmias
+0.01
Hypertension with complications and secondary hypertension
+0.01
Implant, device or graft related encounter
+0.01
Diabetes mellitus, Type 2
+0.01
Administration and transfusion of bone marrow, stem cells, pancreatic islet cells, and t-cells
+0.01
Private
+0.01
Transfusion of blood and blood products
+0.01
Fever
+0.01
Acute and unspecified renal failure
+0.01
Length of Stay
+0.01
Disorders of lipid metabolism
+0.01
Adverse effects of drugs and medicaments, initial encounter
+0.01
Resident
+0.01
Conditions due to neoplasm or the treatment of neoplasm
+0.01
Chronic kidney disease
+0.01
Nonrheumatic and unspecified valve disorders
+0
Sum of 231 other features
+0.1
0.00
0.02
0.04
0.06
0.08
0.10
mean(|SHAP value|)

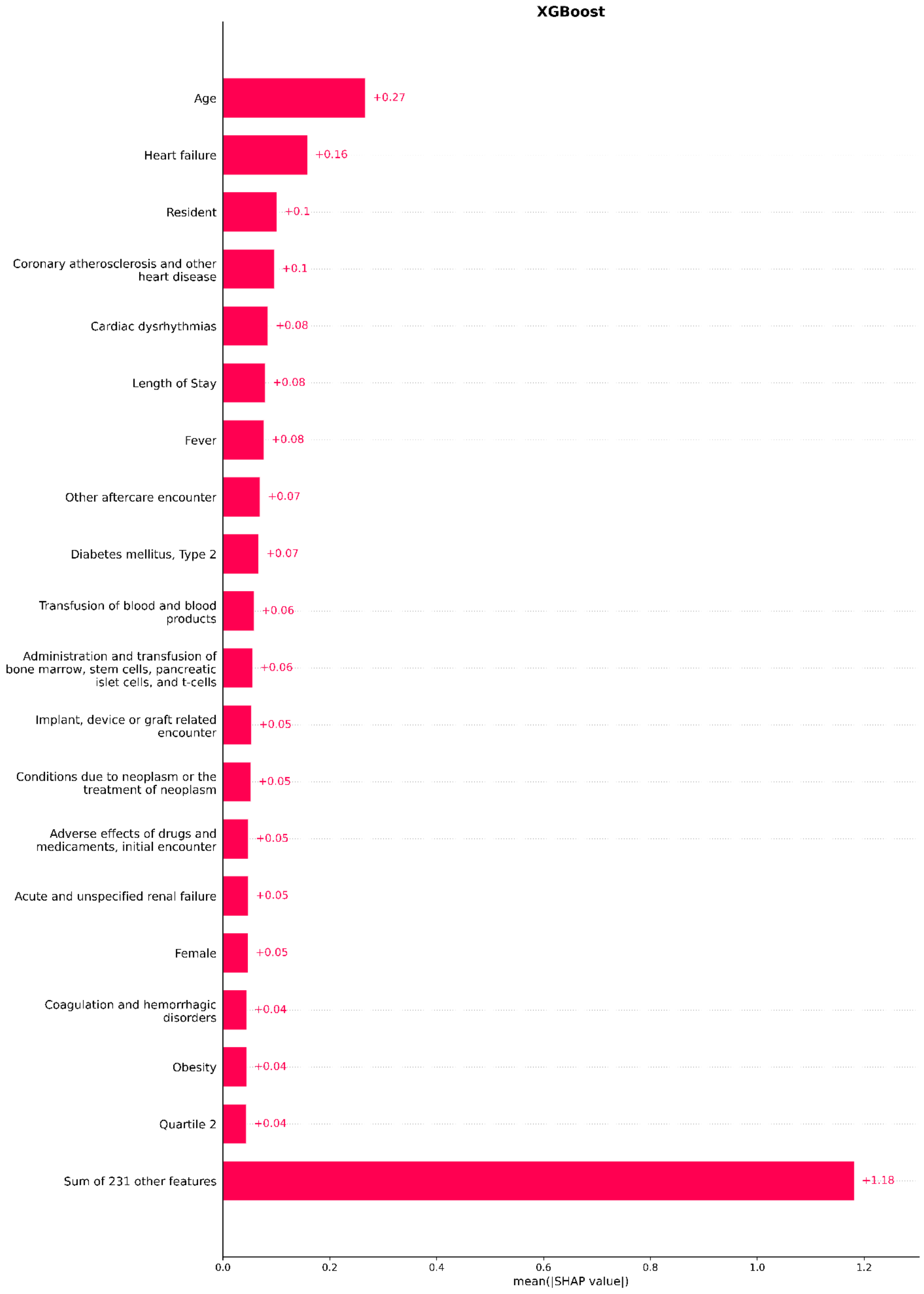
XGBoost
Age
+0.27
Heart failure
+0.16
Resident
+0.1
Coronary atherosclerosis and other heart disease
+0.1
Cardiac dysrhythmias
+0.08
Length of Stay
+0.08
Fever
+0.08
Other aftercare encounter
+0.07
Diabetes mellitus, Type 2
+0.07
Transfusion of blood and blood products
+0.06
Administration and transfusion of bone marrow, stem cells, pancreatic islet cells, and t-cells
+0.06
Implant, device or graft related encounter
+0.05
Conditions due to neoplasm or the treatment of neoplasm
+0.05
Adverse effects of drugs and medicaments, initial encounter
+0.05
Acute and unspecified renal failure
+0.05
Female
+0.05
Coagulation and hemorrhagic disorders
+0.04
Obesity
+0.04
Quartile 2
+0.04
Sum of 231 other features
+1.18
0.0
0.2
0.4
0.6
0.8
1.0
1.2
mean(|SHAP value|)

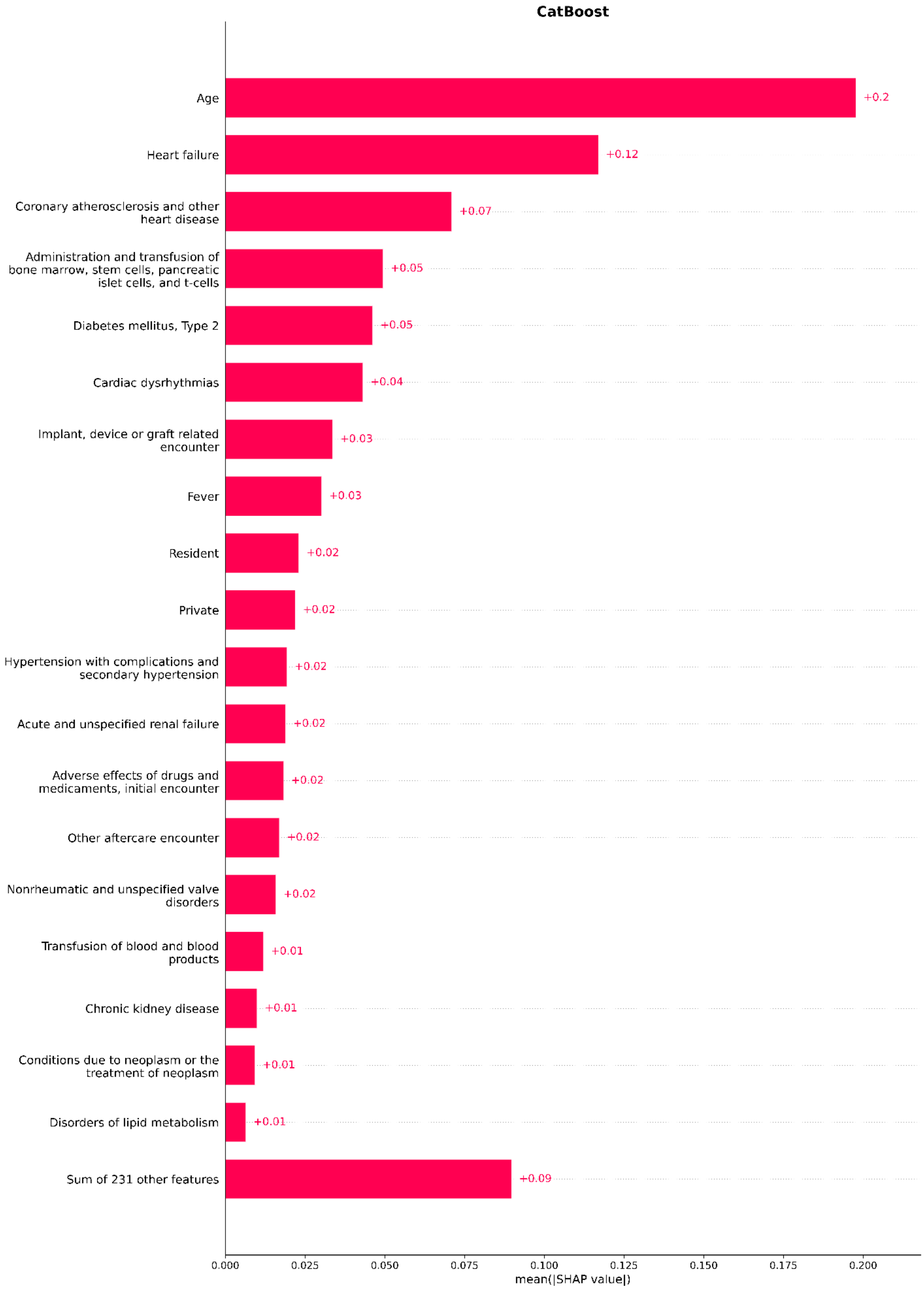
CatBoost
Age
+0.2
Heart failure
+0.12
Coronary atherosclerosis and other heart disease
+0.07
Administration and transfusion of bone marrow, stem cells, pancreatic islet cells, and t-cells
+0.05
Diabetes mellitus, Type 2
+0.05
Cardiac dysrhythmias
+0.04
Implant, device or graft related encounter
+0.03
Fever
+0.03
Resident
+0.02
Private
+0.02
Hypertension with complications and secondary hypertension
+0.02
Acute and unspecified renal failure
+0.02
Adverse effects of drugs and medicaments, initial encounter
+0.02
Other aftercare encounter
+0.02
Nonrheumatic and unspecified valve disorders
+0.02
Transfusion of blood and blood products
+0.01
Chronic kidney disease
+0.01
Conditions due to neoplasm or the treatment of neoplasm
+0.01
Disorders of lipid metabolism
+0.01
Sum of 231 other features
+0.09
0.000
0.025
0.050
0.075
0.100
0.125
0.150
0.175
0.200
mean(|SHAP value|)

Figure 2S. Partial dependence plots of Age (A) and Length of stay (B)

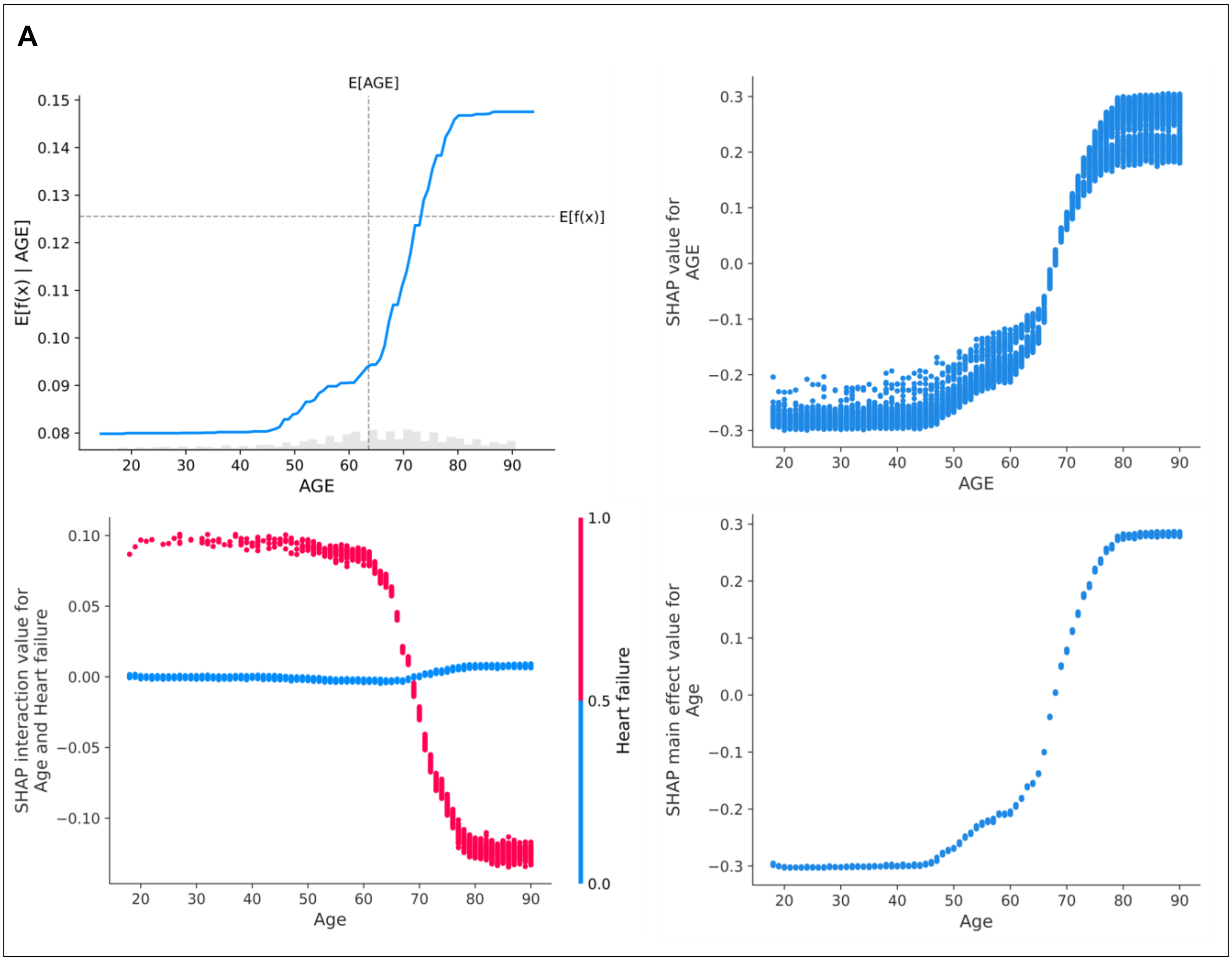

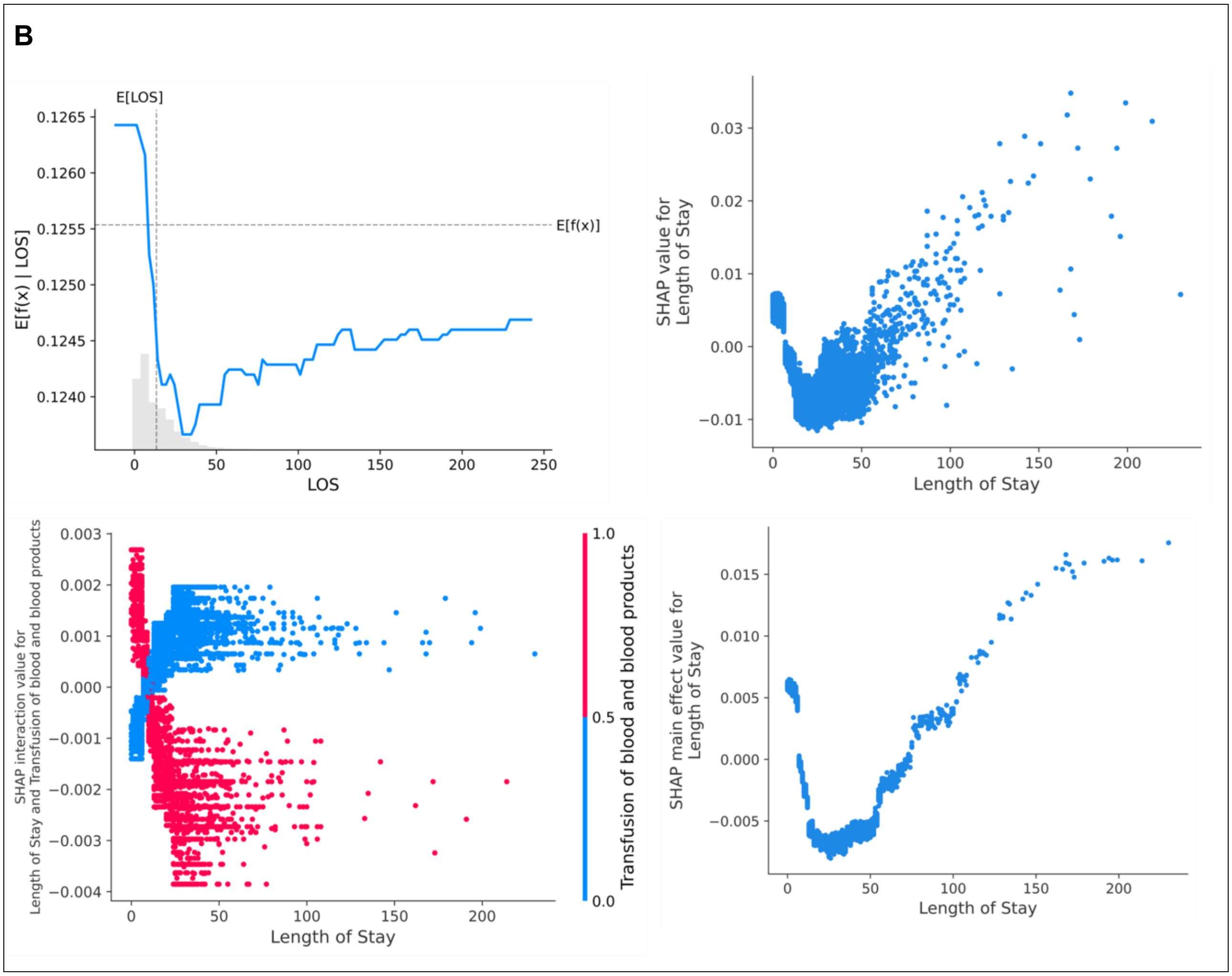
B
E[LOS]
E[f(x)]
E[f(x) | LOS]
LOS
SHAP value for Length of Stay
Length of Stay
SHAP interaction value for Length of Stay and Transfusion of blood and blood products
Transfusion of blood and blood products
SHAP main effect value for Length of Stay

Figure 3S. Predicted log odds quantiles for the study population

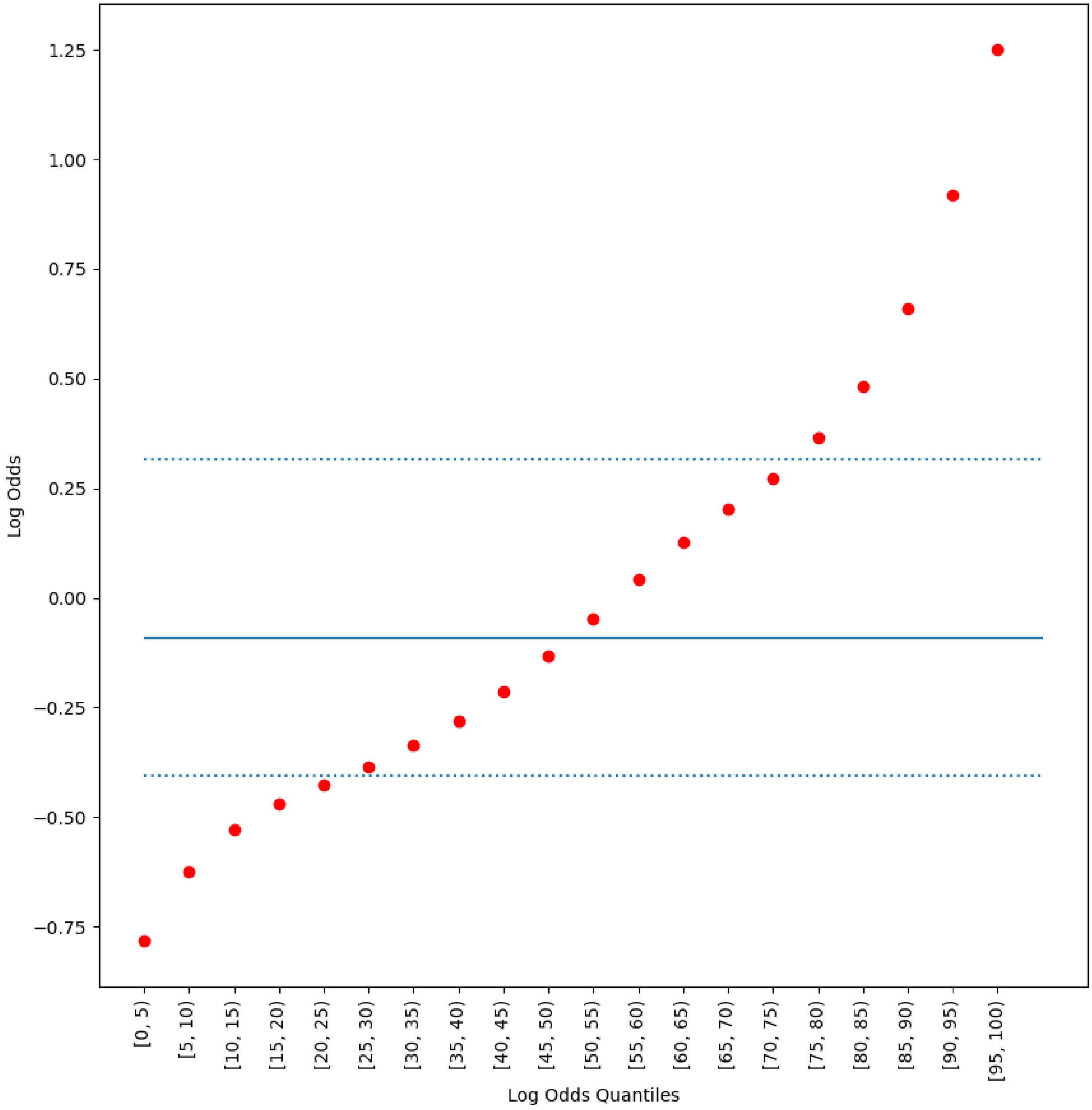

Supplement: Supplemental material - Machine Learning-Based Prediction of Unplanned Readmission Due to Major Adverse Cardiac Events Among Hospitalized Patients with Blood Cancers [file sj-pdf-1-ccx-10.1177_10732748251332803.pdf]
